# The Six1 oncoprotein downregulates p53 via concomitant regulation of RPL26 and microRNA-27a-3p

**DOI:** 10.1038/ncomms10077

**Published:** 2015-12-21

**Authors:** Christina G. Towers, Anna L. Guarnieri, Doug S. Micalizzi, J. Chuck Harrell, Austin E. Gillen, Jihye Kim, Chu-An Wang, Michael U.J. Oliphant, David J. Drasin, Michelle A. Guney, Peter Kabos, Carol A. Sartorius, Aik-Choon Tan, Charles M. Perou, Joaquin M. Espinosa, Heide L. Ford

**Affiliations:** 1 Program in Molecular Biology, University of Colorado, Denver, Anschutz Medical Campus, 12800 East 19th Avenue, Aurora, Colorado 80045, USA; 2 Department of Pharmacology, University of Colorado, Denver, Anschutz Medical Campus, 12800 East 19th Avenue, Aurora, Colorado 80045, USA; 3 Linda Crnic Institute for Down Syndrome, University of Colorado, Denver, Anschutz Medical Campus, Aurora, Colorado 80045, USA; 4 Department of Molecular, Cellular and Developmental Biology, University of Colorado, Boulder, Boulder, Colorado 80203, USA; 5 Howard Hughes Medical Institute, 4000 Jones Bridge Road, Chevy Chase, Maryland 20815-6789, USA; 6 Department of Genetics, University of North Carolina at Chapel Hill, Lineberger Comprehensive Cancer Center, Chapel Hill, North Carolina 27599, USA; 7 Department of Pathology and Laboratory Medicine, University of North Carolina at Chapel Hill, Lineberger Comprehensive Cancer Center, Chapel Hill, North Carolina 27599, USA; 8 Department of Medicine, Division of Medical Oncology, University of Colorado, Denver, Anschutz Medical Campus, 12801 East 17th Avenue, Aurora, Colorado 80045, USA; 9 Integrated Physiology Program, Division of Reproductive Sciences, University of Colorado, Denver, Anschutz Medical Campus, 12800 East 19th Avenue, Aurora, Colorado 80045, USA; 10 Department of Pathology, University of Colorado, Denver, Anschutz Medical Campus, 12801 East 17th Avenue, Aurora, Colorado 80045, USA

## Abstract

TP53 is mutated in 50% of all cancers, and its function is often compromised in cancers where it is not mutated. Here we demonstrate that the pro-tumorigenic/metastatic Six1 homeoprotein decreases p53 levels through a mechanism that does not involve the negative regulator of p53, MDM2. Instead, Six1 regulates p53 via a dual mechanism involving upregulation of microRNA-27a and downregulation of ribosomal protein L26 (RPL26). Mutation analysis confirms that RPL26 inhibits miR-27a binding and prevents microRNA-mediated downregulation of p53. The clinical relevance of this interaction is underscored by the finding that Six1 expression strongly correlates with decreased RPL26 across numerous tumour types. Importantly, we find that Six1 expression leads to marked resistance to therapies targeting the p53–MDM2 interaction. Thus, we identify a competitive mechanism of p53 regulation, which may have consequences for drugs aimed at reinstating p53 function in tumours.

## Abstract (web summary)

p53 is a tumour suppressor that is mutated in a large number of cancers and its expression is controlled largely by the ubiquitin ligase MDM2. Here, the authors show that the homeoprotein, Six1, can regulate p53 in an MDM2- independent manner via regulation of miR-27a and the RNA binding protein, RPL26.

## Graphical abstract

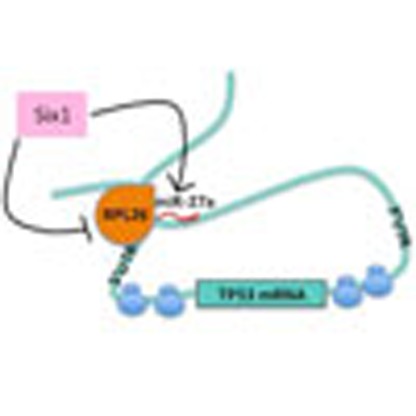

P53 is considered the guardian of the genome because it protects cells from physiological stress, inducing the expression of genes that lead to cell cycle arrest, apoptosis, DNA repair and/or altered metabolism. Accordingly, p53 is mutated in ∼50% of all human tumours, and its function is compromised in a large majority of the remainder^1^. As targeted therapies are being developed to reinstate p53 function in tumours, it is imperative that we understand the underlying mechanisms by which it is regulated.

The most prevalent mechanism of p53 regulation involves the MDM2 protein, an E3 ubiquitin ligase that facilitates rapid polyubiquitination and proteasomal degradation of p53. When MDM2 binds p53, it not only targets p53 for degradation but also occludes the N-terminal alpha-helix of p53, preventing its interaction with transcriptional co-activators and inhibiting its transactivation function. The P53–MDM2 balance is tightly regulated, as MDM2 is a downstream target of p53, creating a negative-feedback loop. In addition, a host of post-translational modifications occur on p53 and MDM2 in response to changes in the cellular environment. These modifications can promote or block p53–MDM2 association^2^. DNA damage is a well-characterized cellular stressor that induces phosphorylation of p53 and MDM2. Phosphorylation in response to DNA damage inhibits the p53/MDM2 interaction, thereby stabilizing p53 and enabling its activation of downstream target genes to regulate tumour suppresson^3^. Thus, the p53–MDM2 association is a critical mechanism of p53 regulation, and increased MDM2 can lead to tumorigenesis^2^. In fact, therapies are currently being developed to target the p53–MDM2 interaction as a means to reinstate p53 function.

Recently, translational regulation of p53 has been shown to affect p53-mediated tumour suppression. Several molecules have been implicated in regulating p53 protein synthesis including RNA-binding proteins (RBPs)^4^,^5^,^6^, translation initiation factors^7^, MDM2 (ref. 8) and p53 itself^9^. In particular, binding of the ribosomal protein L26 (RPL26) to the p53-untranslated regions (UTRs) has been shown to require a double-stranded region of RNA (dsRNA) formed by the 5′- and 3′-UTRs of the p53 messenger RNA (mRNA). This binding leads to increased p53 translation, resulting in higher protein levels and an increase in p53-mediated apoptosis^10^,^11^. MicroRNAs (miRNAs) have also been implicated in post-translational regulation of p53, but only a handful have been shown to directly target the 3′-UTR of p53 (ref. 12). These studies highlight the importance of mechanisms of p53 regulation outside of protein turnover.

The homeodomain containing transcription factor, Six1, is an important developmental regulator that controls cell migration, invasion and proliferation in progenitor cell populations, and is not expressed in most normal adult tissues^13^. Six1 is re-expressed in many cancers including, but not limited to, breast, ovarian, colorectal and hepatocellular carcinoma^14^, where it promotes many of the same properties that it regulates during development. Our laboratory has demonstrated that Six1 mediates tumour initiation, growth and metastasis in mouse models of breast cancer, likely in part through its ability to induce lymphangiogenesis, epithelial-to-mesenchymal transition and tumour initiating cell characteristics. The molecular pathways that mediate Six1-induced phenotypes include VEGF-C upregulation, as well as activation of extracellular signal-regulated kinase (ERK) and transforming growth factor-β (TGFβ) signalling, with TGFβ being regulated in part by Six1 mediated induction of the miR-106b-25 cluster^15^,^16^,^17^,^18^,^19^.

In this study, we demonstrate that the oncoprotein, Six1, downregulates p53. This regulation of p53 by Six1 has important implications for therapies currently in development to stabilize wild-type (WT) p53, such as Nutlin-3 therapies, which we show are ineffective in cells overexpressing Six1. We further show that Six1 decreases the level of p53 protein via simultaneous downregulation of RPL26 and upregulation of miRNA-27a-3p (miR-27a), thus uncovering a competitive mechanism of p53 control working through its UTRs. This mechanism of p53 regulation provides critical insight into how tumours can still inactivate a key tumour suppressive pathway in the absence of p53 mutation. Further, our findings uncover additional oncogenic functions for the developmental regulator Six1.

## Results

### Six1 decreases p53 protein levels and downstream signalling

**Figure 1:**
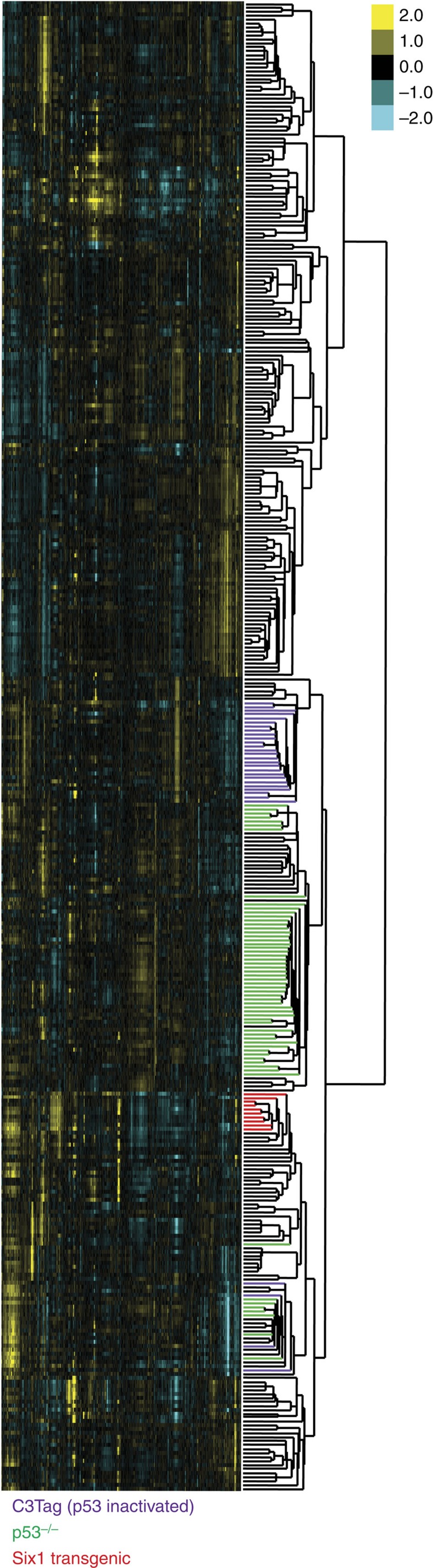
Six1 transgenic tumours are genetically similar to GEMMs with p53 dysfunction. Gene expression microarrays from 377 GEMMs were hierarchically clustered based on an unbiased list of 11,868 gene probes. The dendrogram was coloured based on tumours with deficient p53 (C3Tag tumours (purple), p53null tumours (green)) and on Six1 tumours (red) and those with intact p53 function (black). Six1 transgenic tumours clustered on the same side of the dendrogram as all of the p53-deficient tumours.

Our laboratory previously generated a transgenic mouse model, whereby misexpression of human Six1 in the mouse mammary epithelium induces tumours of multiple histologic subtypes^19^. To determine the molecular profile of these Six1 driven tumours, we performed microarray analysis on 10 Six1-induced mammary tumours, encompassing the spectrum of histologic subtypes. Hierarchical clustering analysis was then performed along with 377 tumours isolated from numerous genetically engineered mouse models (GEMMs) that had previously been analysed^20^. Unbiased examination of all gene probes present (11,868) demonstrate that the Six1 transgenic tumours display a positive node correlation with all of the p53-deficient tumours, clustering on the same side of the dendrogram (Fig. 1).

**Figure 2:**
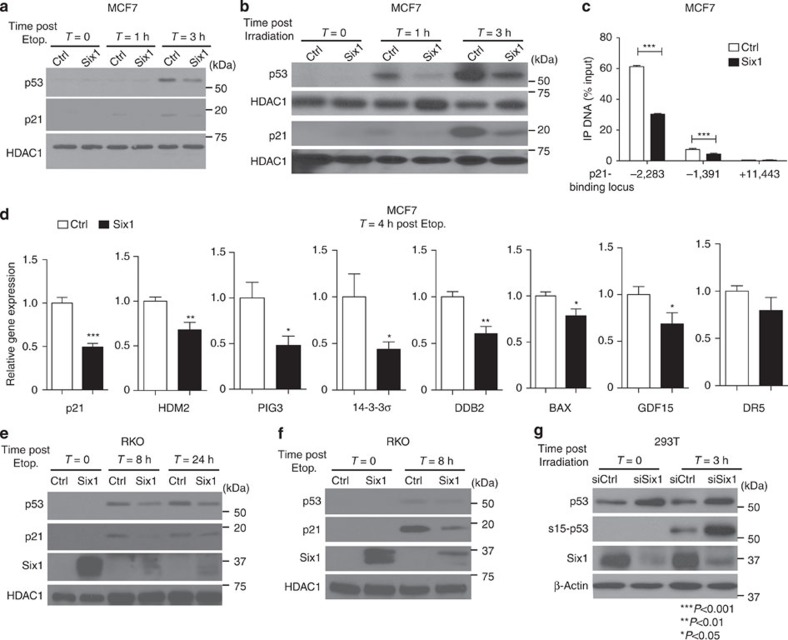
Six1 decreases p53 protein levels and inhibits its downstream signalling. (**a**,**b**) Western blot analyses performed on membranes containing nuclear lysates (NLs) collected from MCF7-Six1 or MCF7-Control (Ctrl) cells that had been treated with (**a**) etoposide (10 μM) or (**b**) ionizing X-irradiation (20 Gy) for indicated time periods. (**c**) ChIP analysis, in MCF7-Ctrl and Six1 cells, of the p21 promoter 4 h after etoposide treatment (10 μM) with a p53 antibody. ChIP-enriched DNA was quantified by real-time PCR analysis using amplicons surrounding the distal (−2,283) and proximal (−1,391) p53 consensus sequences as well as a non-p53-binding site (+11,443). Analysis of variance (ANOVA) on mean±s.d. of triplicate samples for a representative experiment (of two experiments). (**d**) qRT–PCR reveals that overexpression of Six1 in MCF7 cells leads to a decrease in etoposide (10 μM) induced p53 target gene mRNA expression as compared with Ctrl cells. Gene expression is normalized to 18 s. *T*-test on mean±s.d. of triplicate samples for a representative experiment (of ≥2 experiments). (**e**–**f**) Western blot analyses performed on membranes containing NL that were isolated from RKO cells treated with etoposide (10 μM) for the indicated time periods that either (**e**) transiently or (**f**) stably overexpress Six1 or an empty vector (Ctrl). (**g**) Western blot analyses performed on whole-cell lysates from 293T cells transfected with an siRNA pool against Six1 or a non-targeting siRNA pool (siNT). Lysates were made 0 and 3 h after ionizing radiation (5 Gy).

On the basis of the similarity in the expression profiles between the Six1 transgenic tumours and p53-inactivated tumours, we investigated the relationship between Six1 and p53. To determine whether p53 is downregulated by Six1, leading to alterations in p53-mediated signalling, we assessed its expression in MCF7 breast cancer cells engineered to overexpress Six1 (ref. 21), and known to contain WT p53. Importantly, the level of Six1 overexpression is moderate, and well below the level of endogenous Six1 in a number of other cancer cell lines, highlighting that the exogenous expression of Six1 in this system is still within a physiologically relevant range (Supplementary Fig. 1). Western blot analysis performed after induction of DNA double-strand breaks with etoposide shows that stable overexpression of Six1 leads to a decrease in nuclear p53 protein levels, as well as to its well known downstream target p21, in multiple clonal isolates (Fig. 2a, Supplementary Figs 2 and 3 show maintenance of Six1 overexpression in the presence of etoposide). For ease of presenting the data, only one control and Six1 clone is shown after this point throughout the manuscript, although at least two sets of clones were tested for each experiment with similar results. Similarly, we observed a decrease in p53 protein and p21 protein and mRNA expression in response to ionizing radiation-induced DNA damage in the presence of Six1 (Fig. 2b; Supplementary Fig. 4).

To verify that Six1-mediated regulation of p21 was indeed through p53 regulation, we performed chromatin immunoprecipitation followed by quantitative real-time PCR (ChIP–qPCR) to investigate the amount of p53 bound to known p53 consensus sequences within the p21 promoter. Indeed, we found that compared with Ctrl cells, MCF7-Six1 cells have decreased p53 protein bound to both the strong distal-binding site (−2,283) and the proximal, weaker binding site (−1,391) of the p21 promoter^22^, whereas no binding is found at a non-specific site in either case (Fig. 2c).

To confirm that Six1 more widely impinges on signalling downstream of p53, we assessed the expression levels of several additional direct targets of p53 (ref. 23). As expected, Six1 overexpression leads to a decrease in the expression of a number of p53 target genes^23^ in the presence of DNA damage (Fig. 2d; Supplementary Fig. 4). Finally, we examined whether Six1 could regulate p53 in other breast cell line contexts, and found that it also regulates p53 levels, both in the absence and presence of DNA damage, in the MCF12A immortalized breast cell line, which contains wt p53 (Supplementary Fig. 5).

To test the ability of Six1 to decrease p53 levels in other cancer types where it has been implicated^24^, we overexpressed Six1, either transiently or stably, in RKO colon cancer cell lines, which contain wt p53. As was observed in the breast cell lines, the presence of Six1 leads to reduced p53 and its downstream target, p21, in response to etoposide-induced DNA damage (Fig. 2e–f).

Because the above studies were carried out with Six1 overexpression, we further performed knockdown (KD) experiments to determine whether endogenous Six1 controls p53 levels. Transient Six1 KD in 293T cells, an embryonic kidney line transformed with SV40 large T antigen, in which p53 function is altered but its regulation can still be examined, leads to an increase in p53 levels and serine 15 phosphorylated p53 (Fig. 2g). Similarly, efficient knockdown of Six1 in HCT-116 colon cancer cells (which contain wt p53) (HCT-shSix1-1) led to an increase in both p53 and p21 protein levels (Supplementary Fig. 6a,b). Furthermore, p53 target gene^23^ mRNA expression is increased in this context (Supplementary Fig. 6c). Similar to what was observed in the MCF12A system, we demonstrate that Six1 regulates p53 expression, as well as the expression of downstream targets, in the absence of DNA damage in HCT-116 cells.

As a third model to assess whether endogenous Six1 is able to control p53, we performed KD of Six1 in the mouse mammary carcinoma 66Cl4 cell line (confirmed to contain wt p53 by sequencing) and observed that an efficient KD of Six1 leads to an increase in phospho-p53 (Supplementary Fig. 6d,e). Microarray analysis on RNA isolated from 66Cl4 cells with and without Six1 KD followed by Kyoto Encyclopedia of Genes and Genomes pathway analysis demonstrated that p53 target genes (those highlighted in red) are increased upon Six1 KD (Supplementary Fig. 6f). Taken together, these data demonstrate that p53, as well as signalling downstream of p53, is altered in response to Six1 in numerous normal and tumorigenic contexts.

Because Six1 is a homeodomain containing transcription factor, we asked whether it regulates p53 via a direct transcriptional mechanism. To perform these studies, and other mechanistic studies, we utilized our Six1 overexpression rather than KD systems. This choice was made because Six1 is aberrantly overexpressed in cancer, and the lines in which we overexpressed Six1 did not show expression levels higher than those observed in cancer cell lines that endogenously express Six1 (Supplementary Fig. 1). Surprisingly, expression of Six1 did not mediate any changes in the levels of the p53 transcript up to 3 h post treatment with ionizing radiation (Supplementary Fig. 7a). We then examined localization of the p53 protein in response to Six1 overexpression. Although we observed an overall decrease in both total p53 and s-15 phosphorylated p53 protein levels, the cellular localization of p53 in response to Six1 expression was not altered (Supplementary Fig. 7b).

### Six1 regulates p53 independent of MDM2

**Figure 3:**
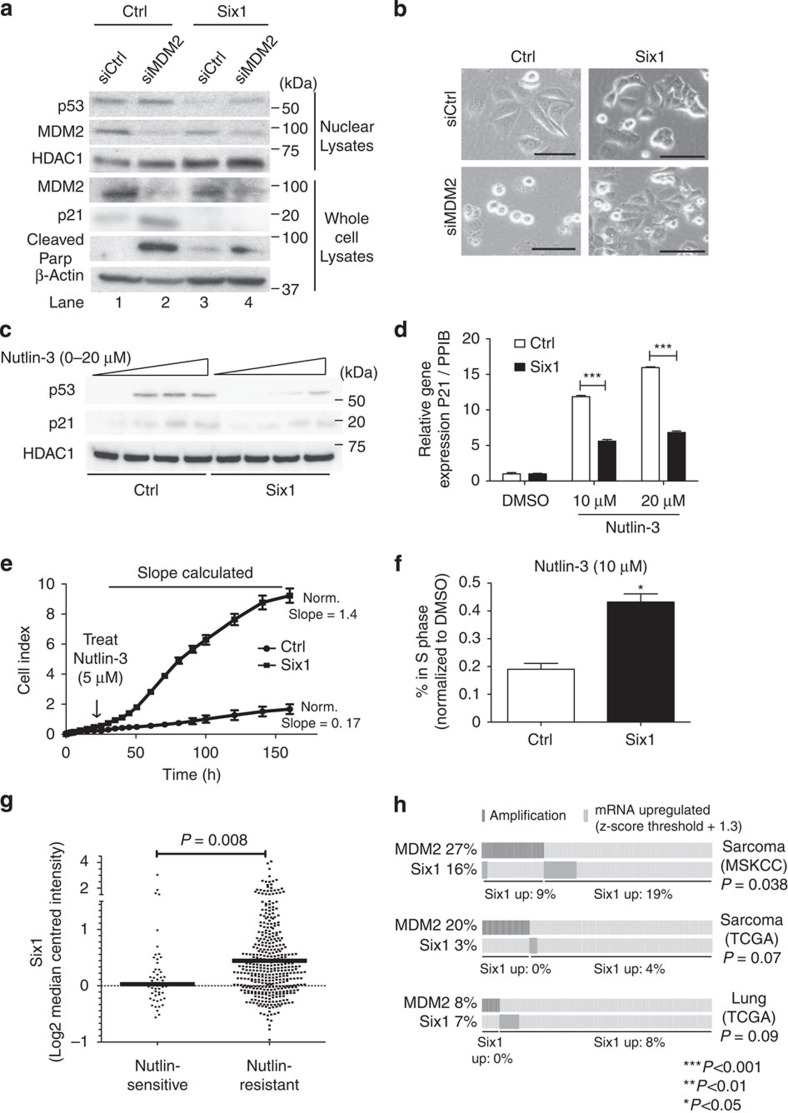
Six1 downregulates p53 independent of MDM2 and induces resistance to MDM2-targeted therapies. In MCF7-Ctrl and Six1 cells, (**a**) western blot analysis was performed on whole-cell lysates (WCLs) and nuclear lysates (NLs) 24 h after transfection with an siRNA pool against MDM2 or an siNT. (**b**) Bright-field images taken 24 h after transfection with an siRNA pool against MDM2. Scale bar, 100 μm (original magnification × 200). (**c**) Western blot analysis on NL collected 3 h after treatment with Nutlin-3. (**d**) qRT–PCR in cells collected 4 h after treatment with Nutlin-3. Gene expression is normalized to PPIB. Analysis of variance (ANOVA) on mean±s.d. of biological triplicates for a representative experiment (of >3 experiments). (**e**) xCELLigence cell proliferation assay. Cells were plated in triplicate to equal confluence and treated with Nutlin-3 (5 μM) and impedance was measured every 30 min for 160 h. The DMSO-normalized slope of the growth curves was calculated over the indicated period of exponential growth. Data shown as mean ±s.d. of triplicate samples for a representative figure (of three experiments). (**f**) BrdU incorporation assay to measure cell cycle distribution 24 h after treatment with Nutlin-3 (10 μM) or DMSO. The data shown are normalized to the DMSO-treated condition. ANOVA on mean±s.d of biological duplicates from two experiments. (**g**) *T*-test on a publicly available gene expression data set of cancer cell lines from multiple tumour types via Oncomine shows that cell lines resistant to Nutlin-3 (IC_50_>5 μM) have increased expression of Six1. (**h**) One-tailed *χ*^2^-test performed on publicly available patient data sets via cBioPortal^68^,^69^ examining the mutual exclusivity between MDM2 gene amplification and Six1 mRNA overexpression (*z*-score threshold for Six1 expression is +1.3).

Under normal cellular conditions, p53 is tightly regulated by the E3 ubiquitin ligase, MDM2, which targets p53 for degradation^2^. Thus, we investigated whether Six1 may upregulate MDM2, resulting in p53 downregulation. However, we found that instead of increasing MDM2 levels, Six1 overexpression in MCF7 cells resulted in MDM2 downregulation (Fig. 3a; Supplementary Fig. 8a). Since MDM2 is transcriptionally regulated by p53 as part of a negative-feedback loop^25^, this result suggests that Six1 is not regulating MDM2 to influence p53, but is rather regulating p53, which then feeds back on the transcription of MDM2. Similarly, we observed a modest downregulation of MDM2 in the RKO system in response to Six1 overexpression, and importantly, did not see Six1-induced upregulation of MDM2 (Supplementary Fig. 8b). These data further demonstrate that upregulation of MDM2 is not the mechanism by which Six1 downregulates p53.

The above results led us to hypothesize that Six1 can decrease p53 protein levels via a novel, MDM2-independent mechanism. To test this hypothesis, we used an short interfering RNA (siRNA) pool to transiently (KD) MDM2 in MCF7-Ctrl and Six1 overexpressing cells. KD of MDM2 causes an increase in p53 and p21 in the Ctrl cells (Fig. 3a). While MDM2 KD also causes an increase in p53 levels in the MCF7-Six1 cells as compared with the Six1 cells with non-targeting siRNA, the levels of p53, and its target p21, remain lower in the Six1 overexpressing cells than in the Ctrl cells, with or without MDM2 KD (Fig. 3a, lanes 2 and 4). Thus, the ability of Six1 to decrease p53 is not dependent on MDM2. Importantly, similar results were seen in RKO-Ctrl and Six1 overexpressing cells, whereby siRNA KD of MDM2 does not rescue the ability of Six1 to decrease p53 protein expression (Supplementary Fig. 8b).

In response to MDM2 KD, the MCF7-Ctrl cells significantly rounded up, whereas the MCF7-Six1 cells continued to appear healthy (Fig. 3b). The morphological changes observed in MCF7-Ctrl cells were reminiscent of cell death, known to be induced by p53. We thus measured the induction of a cleavage product of poly (ADP-ribose) polymerase (PARP) as an assessment of cell death. Indeed, we observed that Six1 expression leads to a marked decrease in the induction of PARP cleavage in response to MDM2 KD (Fig. 3a). These results strongly suggest that Six1 overexpression allows cells to survive loss of MDM2, likely due to its ability to maintain low p53 levels even in the absence of MDM2 regulation.

### Six1 causes resistance to MDM2-targeted drugs

Because Six1 downregulates p53 in an MDM2-independent manner, we hypothesized that tumours with high expression of Six1 would be resistant to MDM2-targeted therapies. In line with this hypothesis, MCF7-Six1 cells treated with increasing doses of the MDM2-targeted therapy, Nutlin-3, maintain lower levels of p53, and its target, p21, when compared with MCF7-Ctrl cells at 3 and 24 h after treatment (Figs 3c,d; Supplementary Fig. 9a,b). Importantly, Six1 expression is not altered by Nutlin-3 treatment (Supplementary Fig. 9c). In addition, MCF7-Six1 cells have a marked increase in cell index (an indirect measure of cell number) in the xCELLigence assay, as compared with the MCF7-Ctrl cell lines after Nutlin-3 treatment (Fig. 3e). To quantify the difference in Nutlin-3 response between MCF7-Six1 and Ctrl cells, we measured the dimethylsulphoxide (DMSO)-normalized slope of the growth curves after Nutlin-3 treatment, and found that the presence of Six1 increases the index rate by eight-fold (*P*<0.0001). Furthermore, when we performed the xCELLigence assay over a range of doses, we observed a significant increase in the half-maximal inhibitory concentration (IC_50_) with Six1 overexpression (IC_50_=65 μM) as compared to the Ctrl cells (IC_50_=3.06 μM). To validate these data using different methodologies, we performed bromodeoxyuridine (BrdU) incorporation, MTS and colony formation assays in MCF7 cells, which all demonstrate that cells treated with Nutlin-3 are capable of continued growth in the presence of Six1 (Fig. 3f; Supplementary Fig. 9d,e). To confirm that these effects are generalizable to MDM2-targeted therapies, we treated our MCF7-Ctrl and Six1 overexpressing cells with JNJ26854165 (Serdementan), another small molecule that binds to the p53-binding pocket of MDM2. Similar to Nutlin-3, Six1 overexpression induces resistance to JNJ26854165 (Supplementary Fig. 9f,g). Finally, to examine whether Six1 regulates p53 via an MDM2-independent mechanism in other cell systems, we repeated the xCELLigence experiments in MCF12A cells with and without Six1 overexpression, and found that, similar to what was observed in the MCF7 system, overexpression of Six1 leads to Nutlin-3 resistance in MCF12A cells (Supplementary Fig. 9h,i).

To determine whether Six1 expression correlates with response to Nutlin-3 therapy over a wide spectrum of tumour types in addition to breast cancer, we analysed public data sets measuring drug resistance across 451 human cancer cell lines^26^. Figure 3g shows that there is a 10-fold increase in the median Six1 expression in cell lines that are resistant to Nutlin-3. However, increased Six1 expression does not correlate with resistance to other breast cancer therapies, including cisplatin, doxorubicin or paclitaxel, in this same data set, suggesting that it does not induce global drug resistance, but is specific to MDM2-targeted therapies (Supplementary Fig. 10). Taken together, these data demonstrate that Six1 regulates p53 independent of MDM2, thus resulting in resistance to MDM2-targeted therapies.

Finally, because Six1 regulates p53 independent of MDM2, we hypothesized that patient tumours with MDM2 gene amplification may be mutually exclusive from Six1 overexpression. While we found few data sets in which a high percentage of patients had MDM2 gene amplification, in those that did (sarcoma and lung data sets^27^,^28^), we were able to observe a trend towards or significant mutual exclusivity between Six1 overexpression and MDM2 amplification (Fig. 3h). In addition, we saw a similar trend across many other tumour types where patient cohorts were not large enough to reach significant *P* values. These results strongly argue that Six1 mediates p53 downregulation in an MDM2-independent manner in human tumours.

### Six1 does not affect proteasome-mediated degradation of p53

**Figure 4:**
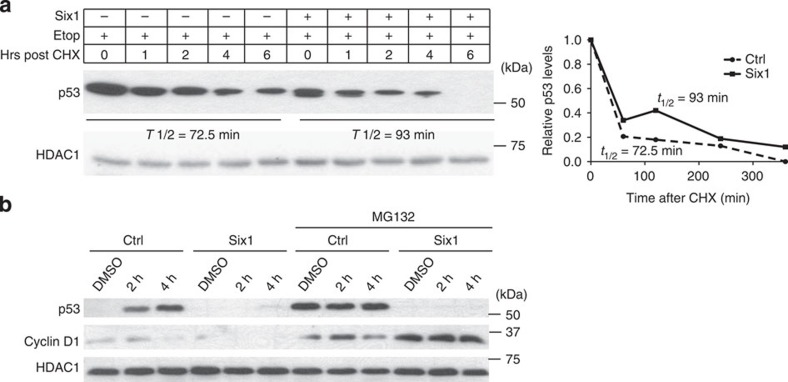
Six1 downregulates p53 independent of proteasome-mediated protein degradation. Nuclear lysates from MCF7-Ctrl and Six1 cells (**a**) treated with etoposide (10 μM) for 2 h were subsequently treated with cycloheximide (20 μM) and collected after indicated periods of time. Right: p53 half-life was calculated by quantification of western blots using Image Lab software. (**b**) MCF7-Six1 and Ctrl cell lines treated with etoposide (10 μM) and MG132 (25 μM) for indicated periods of time. Membranes were probed for Cyclin-D1 to demonstrate that the MG132 used was effective.

Multiple E3 ubiquitin ligases have been shown to target p53 for degradation through the proteasome^29^. To determine whether Six1 is able to downregulate p53 independent of all ubiquitin ligases, we analysed the half-life of the p53 protein in response to Six1 overexpression. MCF7-Ctrl and Six1 lines were treated with cycloheximide (CHX), a potent inhibitor of protein translation. Western blot analysis was then performed at indicated time periods after CHX treatment to measure p53 half-life. Quantification of western blot analysis shows that overexpression of Six1 does not cause a decrease in the degradation time/half-life of the p53 protein after DNA damage (or in the absence of DNA damage; Fig. 4a; Supplementary Fig. 11a). Furthermore, treatment of MCF7-Six1 cells with the proteasome inhibitor, MG132, does not inhibit the ability of Six1 (whose overexpression is maintained with both CHX and MG132 treatment (Supplementary Fig. 11b–e)) to downregulate p53, whereas it is able to stabilize cyclin D1, a known Six1 transcriptional target^30^ (Fig. 4b). Similarly, MG132 treatment in MCF12A-Ctrl and Six1 cells did not influence the ability of Six1 to decrease p53 protein levels (Supplementary Fig. 12). Taken together, these results clearly demonstrate that Six1 does not alter proteasome-mediated degradation of p53, and must therefore regulate p53 independent of the ubiquitin–proteasome pathway.

### Six1 downregulates p53 in part via regulation of miR-27a

**Figure 5:**
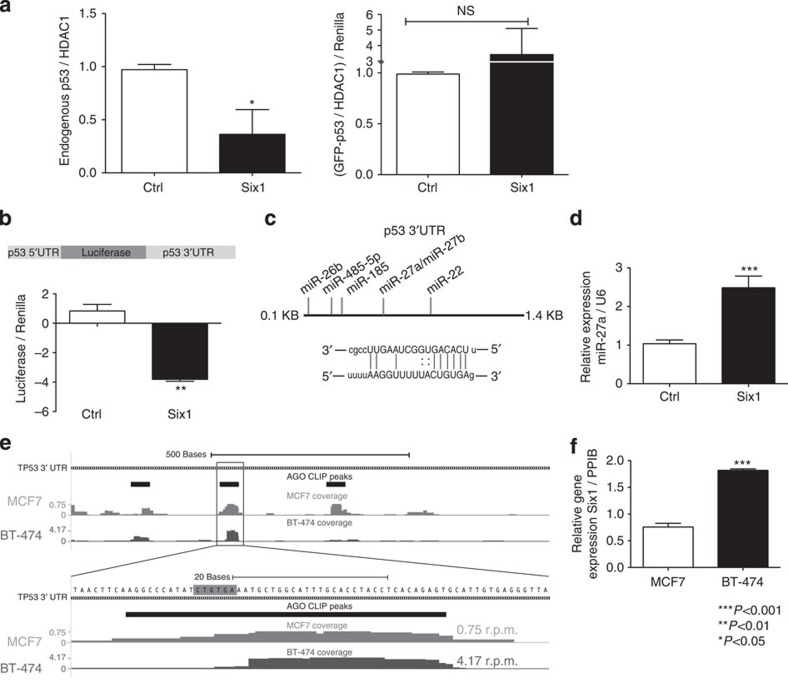
Six1 downregulates p53 via a microRNA-mediated mechanism. (**a**) MCF7-Ctrl and Six1 cells were co-transfected with GFP–p53 and a constitutively active renilla-luciferase construct. After 1 h of etoposide treatment (10 μM), cell pellets were divided to obtain nuclear lysates (NLs) and perform luciferase assays. Endogenous p53 (left) and GFP–p53 bands (right) were quantified and normalized to endogenous HDAC1 levels. Transfected GFP–p53 was additionally normalized to the renilla luciferase values to control for transfection efficiency. Combination of 2 independent experiments, each run with experimental duplicates. (**b**) MCF7-Six1 or Ctrl cells were transfected with a luciferase construct containing 145 base pairs of the p53 5′-UTR and the entire p53 3′-UTR (top), as well as with a constitutively active renilla luciferase construct. *T*-test on mean±s.d of biological triplicates for a representative experiment (>3 experiments). (**c**) Top: a diagram of the microRNAs predicted to bind the p53 3′-UTR^31^ that were upregulated by Six1 via a microRNA microarray^18^. Bottom: the predicted binding of miR-27a to the p53 3′-UTR^31^. (**d**) qRT–PCR for mature miR-27a expression in MCF7-Six1 and Ctrl cells. *T*-test on mean±s.d. of biological triplicates for a representative experiment (three experiments). (**e**) The 3′-UTR of p53 showing representative HITS-CLIP coverage in BT-474 cells and MCF7 cells. Statistically significant Ago footprints (peaks) are shown above the coverage. Coverage scale (left) is in reads per million mapped reads (r.p.m.). The sequence complementary to the miR-27a-3p seed site is highlighted in grey. (**f**) qRT–PCR for Six1 expression in BT-474 cells as compared with MCF7 cells. *T*-test on mean±s.e.m. of biological triplicates for a representative experiment (two experiments).

UTRs in mRNAs are necessary for miRNA-mediated regulation of protein expression. Thus, we examined whether the UTRs of p53 are required for its regulation by Six1. To this end, we transfected MCF7-Ctrl and Six1 cells with a green fluorescent protein (GFP)-tagged p53 containing the coding sequence of p53, but lacking the 5′- and 3′-UTR sequences. To control for transfection efficiency, the cells were co-transfected with a renilla luciferase plasmid. Following treatment with etoposide to enhance p53 levels, lysates were collected and used in both luciferase assays and western blot analyses to examine the levels of endogenous p53 and exogenous GFP–p53, which are different molecular weights due to the tag on the exogenous p53. We observed that Six1 is able to decrease the endogenous p53 protein, but not the exogenous GFP–p53 that does not contain any UTR sequences (Fig. 5a; Supplementary Fig. 13a). These results demonstrate that Six1 requires the UTRs of p53 to regulate its levels.

To test whether the UTRs of p53 are sufficient to mediate downregulation by Six1, we transfected a luciferase construct containing the 5′- and 3′-UTR of p53 into MCF7 cells with stable overexpression of Six1, as well as in 293T cells with transient overexpression of Six1. In both systems, we found that overexpression of Six1 leads to a decrease in luciferase activity; thus, the UTRs of p53 are sufficient for Six1-mediated p53 regulation (Fig. 5b; Supplementary Fig. 13b).

To investigate whether miRNAs could potentially regulate p53 expression downstream of Six1, we interrogated our previously generated miRNA microarray data set, which was obtained by comparing the miRNA profile of MCF7-Six1 cells with MCF7-Ctrl cells^18^. We identified six miRNAs that were upregulated by Six1 and are predicted to target the p53 3′-UTR^31^ (Fig. 5c). We were unable to verify Six1-mediated upregulation of miR-27b, miR-185, miR-22 or miR-485-5p (could not amplify miR-485-5p) by qRT–PCR using miRNA-specific primers nor were we able to observe Six1-mediated upregulation of already known p53 targeting miRNAs 504 or 125b (Supplementary Fig. 14a–c,e–f). However, we found that two of the Six1-regulated miRNAs predicted to target p53, miR-27a and miR-26b are upregulated by Six1 in MCF7 cells (Fig. 5d; Supplementary Fig. 14d). Although we found that Six1 could upregulate miR-26b, we were unable to demonstrate that miR-26b could downregulate p53, either by luciferase assays or western blot analysis (Supplementary Fig. 14g,h).

The final miR we examined, miR-27a, has been described as an oncoMiR^32^,^33^,^34^,^35^,^36^ in line with its upregulation by Six1. Thus, we first confirmed that miR-27a was also upregulated by Six1 in another system, 293T cells (Supplementary Fig. 15a). We found one predicted binding site for miR-27a in the p53 3′-UTR with perfect base pairing at residues 2–7 (Fig. 5c). In an attempt to validate p53 as a miR-27a target, we co-transfected parental MCF7 or 293T cells with a miR-27a mimic and the p53-UTR luciferase reporter construct, but surprisingly, results from these experiments were inconclusive. Thus, we used a more functional assay to examine whether the miR-27a-binding site within the 3′-UTR of p53 was actually occupied in breast cancer cells. To this end, we interrogated our high-throughput sequencing data in which Argonaute (Ago) was cross-linked to RNA and immunoprecipitated (IP) to isolate miRNA–mRNA–Ago complexes (HITS-CLIP) in multiple breast cancer cell lines^37^. In this data set, we specifically focused on the p53 3′-UTR sequences that were IP with Ago in MCF7 and BT-474 breast cancer cells. A significant enrichment in the sequence corresponding to the miR-27a-binding site in the p53 3′-UTR was seen in both the MCF7 (*q*=1.56e−34) and BT-474 (*q*=4.92e−172) cells (Fig. 5e). Interestingly, the BT-474 cells, which express higher endogenous levels of Six1 than the MCF7 cells (Fig. 5f), had a larger number of reads per million (4.17 r.p.m.) at the miR-27a-binding site when compared with the MCF7 cells (0.74 r.p.m.). We were unable to find any significant enrichment in the sequences corresponding to the miR-26b-binding site in either cell line, further indicating that miR-26b cannot bind the p53 3′-UTR, and that miR-27a may be the more relevant miR downstream of Six1.

Further, when we interrogated mRNA and miRNA profiles in patient data sets, we observed a trend towards (*P*=0.06 and *P*=0.17) a positive correlation between Six1 and miR-27a expression in the normal-like and ERBB2 subtypes of breast cancer, respectively, suggesting that in a context-specific manner, Six1 likely regulates miR-27a in human cancers (Supplementary Fig. 15b)^38^.

The above results suggest that miR-27a can bind to the p53 3′-UTR and, taken together with the fact that Six1 can upregulate miR-27a, strongly suggest that Six1 may regulate p53 through its ability to alter miR-27a levels. However, since we were unable to conclusively demonstrate that miR-27a could regulate the p53 3′-UTR in the context of low Six1 expression (when we exogenously introduced the miR in the absence of Six1 overexpression), we reasoned that Six1 may regulate yet another factor that cooperatively works with miR-27a to inhibit p53 translation.

### Six1 downregulates the ribosomal protein L26

**Figure 6:**
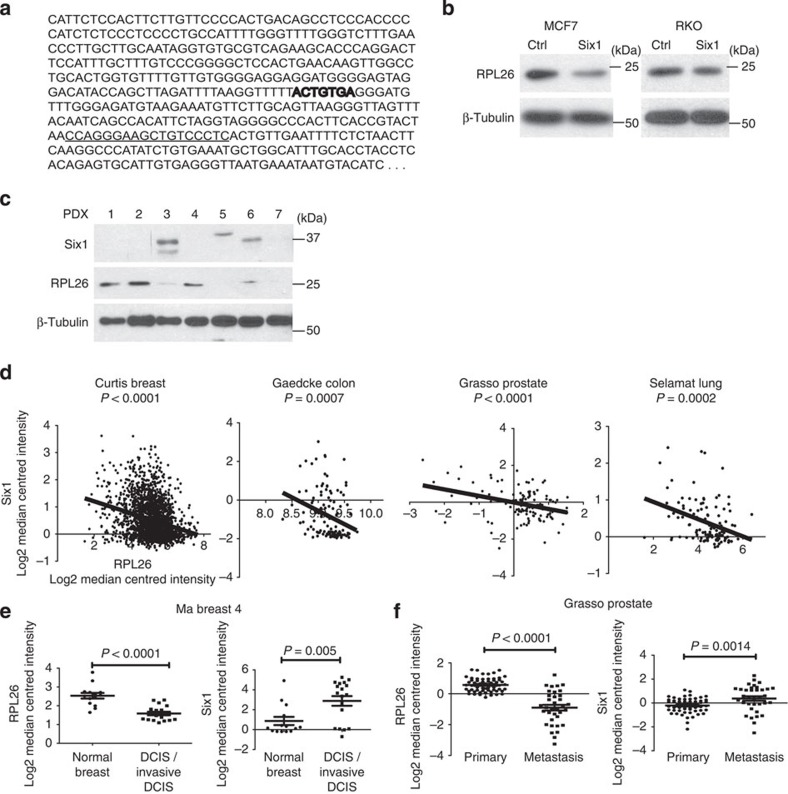
Six1 decreases RPL26 expression. (**a**) A partial sequence of the p53 3′-UTR indicating that the predicted miR-27a-binding site (bold) is 93-bp away from the sequence in the 3′-UTR required for RPL26 binding (underlined). (**b**) Western blot analyses performed using whole-cell lysates (WCLs) collected from MCF7 and RKO cells with stable overexpression of Six1. (**c**) Western blot analysis of WCLs collected from seven PDX samples. (**d**) Linear regression analyses examining the inverse correlation between Six1 and RPL26 expression in patient tumours obtained from the Curtis breast, Gaedcke colon, Grasso prostate and Selemat lung cancer data sets via Oncomine. (**e**) RPL26 and Six1 expression in the Ma breast four data set in DCIS or invasive DCIS as compared with matched normal breast tissue via Oncomine. (**f**) RPL26 and Six1 expression in the Grasso prostate data set comparing primary prostate lesions with metastatic lesions via Oncomine. (**e**,**f**) The data are shown as the mean±s.e.m. and *P* values were calculated using an unpaired two-tailed Student's *T*-test.

To investigate the aforementioned issue, we examined the p53 3′-UTR sequence surrounding the miR-27a-binding site. We found that the sequence of the 3′-UTR that forms a dsRNA that is necessary for binding of a translational regulator of p53, RPL26, is in proximity to the miR-27a-binding site (93 base pairs away; Fig. 6a). This region of dsRNA that forms between the 5′- and 3′-UTRs of p53 is critical for the binding of RPL26 and results in increased p53 protein expression via increased translation^10^,^11^. Thus, we hypothesized that binding of RPL26 may interfere with miR-27a binding to the p53 3′-UTR, and that Six1 may act not only to upregulate miR-27a but also to downregulate RPL26. Indeed, Six1 overexpression in three systems (MCF7, RKO and 293T cells) leads to a marked downregulation of RPL26 (Fig. 6b; Supplementary Fig. 16a). To investigate whether Six1-mediated downregulation of RPL26 is relevant to human cancer, we performed western blot analysis on seven breast cancer patient-derived xenografts (PDXs). Intriguingly, we found that PDXs with elevated Six1 expression often have a lower level of RPL26 protein as compared with PDXs with undetectable levels of Six1 protein (Fig. 6c). We confirmed this regulation in larger patient data sets (on the mRNA level), as Six1 expression significantly inversely correlates with RPL26 expression across multiple tumour types, including breast, colon, prostate, lung, cervical, gastric and renal cancer (Fig. 6d; Supplementary Fig. 16b–h)^28^,^39^,^40^,^41^,^42^,^43^,^44^,^45^,^46^. Similarly, RPL26 and Six1 have a significant inverse correlation with regards to prognosis. Six1 is overexpressed in a plethora of different tumour types and correlates with worsened prognosis^16^,^19^,^47^,^48^. The inverse is true for RPL26, which is decreased in numerous cancers when compared with normal counterparts (Supplementary Table 1)^28^,^39^,^41^,^49^,^50^,^51^,^52^,^53^. In breast cancer, RPL26 expression is decreased in ductal carcinoma *in situ* (DCIS) and invasive DCIS as compared with normal breast tissue. In contrast, in the same data set, Six1 is upregulated in DCIS and invasive DCIS as compared with normal breast tissue (Fig. 6e)^49^. Similar results are seen in prostate cancer, where RPL26 is decreased in the metastatic lesions as compared with the primary tumour, whereas Six1 is increased in the metastatic lesions as compared with the primary tumour (Fig. 6f)^41^. Thus, Six1 and RPL26 strongly inversely correlate in numerous human tumorigenic settings.

### Six1 regulates p53 via regulation of both miR-27a and RPL26

**Figure 7:**
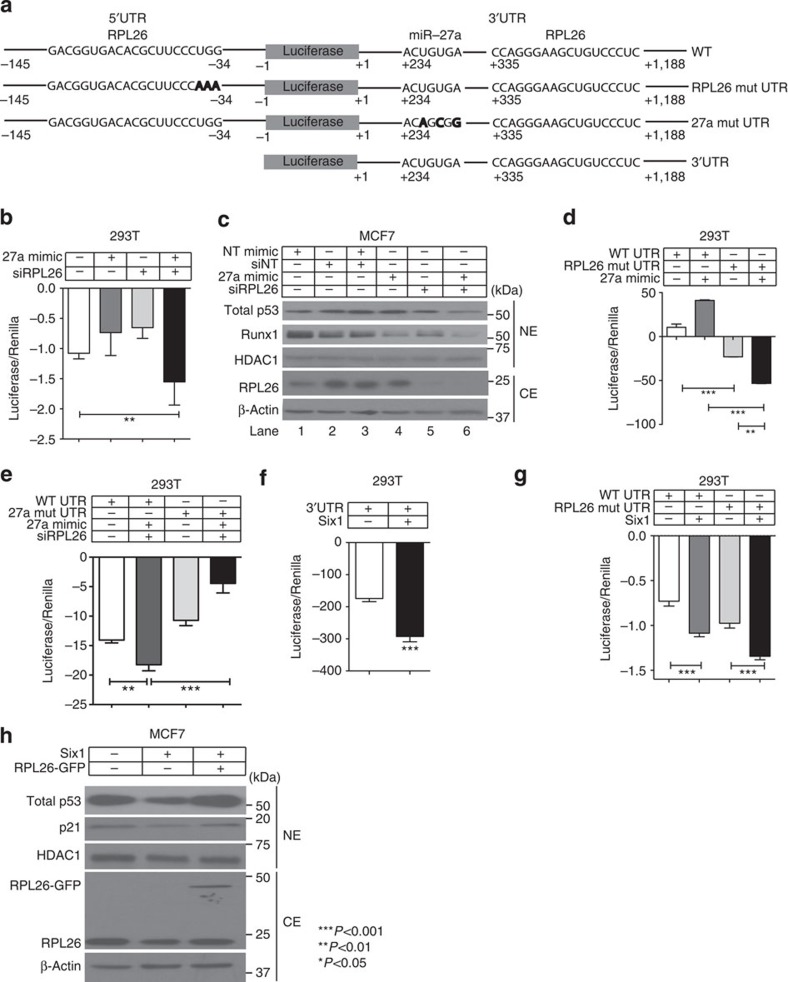
Six1 downregulates p53 via simultaneous upregulation of miR-27a and downregulation of RPL26. (**a**) Depiction of the luciferase constructs used and the mutations made (bold) in the regions necessary for RPL26 binding and miR-27a binding to the p53 UTRs. (**b**) Luciferase assay performed in 293T cells transfected with the WT luciferase vector, miR-27a mimic or a scrambled mimic, and a pooled siRNA against RPL26 (siRPL26) or a control siNT (non-targeting) for 48 h. Analysis of variance (ANOVA) on mean±s.e.m. of biological triplicates for three combined experiments. (**c**) Western bot analysis performed on nuclear lysates and cytoplasmic lysates from MCF7 cells transiently transfected with a miR-27a mimic or a scrambled mimic and siRPL26 or siNT for 72 h. Runx1 levels were examined to demonstrate that the miR-27a mimic was functional. (**d**) Luciferase assay performed in 293T cells 48 h after transfection with a miR-27a or scrambled mimic and with the RPL26 WT or mutant reporter vector. ANOVA on mean±s.d. of biological triplicates for a representative experiment (of three experiments). (**e**) Luciferase assay performed in 293T cells transfected with the p53 WT UTR vector or with the 27a mut UTR vector and with miR-27a or scrambled mimic, and an siRPL26 pool or an siNT. ANOVA on mean±s.d. of biological triplicates for a representative experiment (of three experiments). (**f**) Luciferase assay performed in 293T cells 48 h after transfection with Six1 and the 3′-UTR reporter vector. ANOVA on mean±s.d. of biological triplicates for a representative experiment (of three experiments). (**g**) Luciferase assay performed in 293T cells 48 h after transfection with Six1 and either the WT or RPL26 mutant UTR reporter vectors. ANOVA on mean±s.d. of biological triplicates for a representative experiment (three experiments). (**h**) Western Blot analysis performed on nuclear lysates and cytoplasmic lysates from MCF7-Ctrl and Six1 cells transiently transfected with GFP-RPL26 or GFP. Note: When (−) is used, the correct non-targeting control was transfected in.

Using a series of luciferase reporter constructs, we assessed the ability of RPL26 to inhibit miR-27a from binding to the p53 3′-UTR (Fig. 7a). To specifically test whether miR-27a can decrease luciferase signal from a reporter vector containing the 5′- and 3′-UTRs of p53 (WT reporter) in the absence of RPL26, we co-transfected 293T cells with a miR-27a mimic in the presence or absence of an siRNA pool against RPL26. As shown in Supplementary Fig. 17, we achieved simultaneous overexpression of miR-27a and KD of RPL26. Overexpression of miR-27a causes a significant decrease in luciferase expression, but only in the context of RPL26 KD (Fig. 7b). Similar trends were seen with p53 protein expression in MCF7 cells; overexpression of miR-27a on its own does not cause a decrease in p53 levels when compared with overexpression of a scrambled control (Fig. 7c, lane 1 and 4), yet it does lead to a decrease in a known target of miR-27a, Runx1 (ref. 54). KD of RPL26 causes a slight decrease in p53 levels as compared with a non-targeting siRNA control (Fig. 7c, lanes 2 and 5). However, simultaneous overexpression of miR-27a and KD of RPL26 causes a marked decrease in p53 protein expression compared with cells transfected with a scrambled mimic along with a non-targeting siRNA (Fig. 7c, lanes 3 and 6).

To confirm that RPL26 inhibits miR-27a binding to the p53 3′-UTR, we mutated the critical sequence for RPL26 binding in the 5′-UTR of the reporter luciferase construct, by altering three base pairs that were previously shown to be critical for RPL26 to bind to the p53 mRNA^11^ (Fig. 7a). As expected, in 293T cells, the RPL26 mutant UTR reporter shows a decrease in luciferase signal when compared with the WT reporter (Fig. 7d). Notably, miR-27a decreases luciferase activity only in the context of the RPL26 mutant reporter (Fig. 7d, compare second and fourth lanes), demonstrating that miR-27a can only decrease p53 levels when RPL26 cannot bind to the p53 UTRs. To confirm that miR-27a is binding directly to the p53 3′-UTR, we also mutated three base pairs in the miR-27a-binding site (Fig. 7a), which, as expected, inhibits its ability to decrease the luciferase signal in the context of RPL26 KD in 293T cells (Fig. 7e, compare second and fourth lanes). To demonstrate that Six1-mediated repression of p53 is not solely caused by its ability to downregulate RPL26, but is also enhanced by its ability to upregulate miR-27a, we used a luciferase vector containing only the p53 3′-UTR, resulting in a construct to which RPL26 can no longer bind due to the lack of the 5′-UTR (Fig. 7a). Overexpression of Six1 in 293T cells leads to a decrease in reporter signal even in the absence of the 5′-UTR, indicating that Six1 can mediate a decrease in p53 translation via a mechanism that is separate from its ability to regulate RPL26 (Fig. 7f). More specifically, Six1 maintains its ability to decrease reporter activity when the RPL26 mutant reporter is used, further supporting the notion that Six1 is able to decrease p53 expression via a dual mechanism (Fig. 7g). Finally, addition of exogenous GFP-tagged RPL26 into Six1 overexpressing cells restores p53 levels and leads to an increase in p21 expression (Fig. 7h). On the basis of our findings, we speculate that a full rescue is obtained in the MCF7-Six1 cells solely by reinstating RPL26, due to the fact that RPL26 inhibits miR-27a from binding to the p53 3′-UTR.

## Discussion

**Figure 8:**
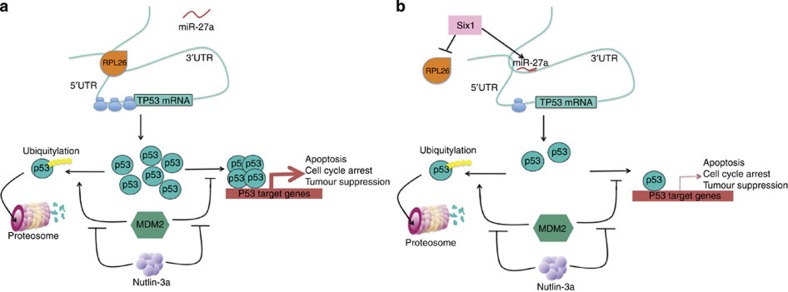
A graphical model. (**a**) A model of p53 regulation in the absence of Six1 whereby RPL26 binds the 5′- and 3′-UTR of p53, leading to an increase in translation and p53 protein expression. The newly translated p53 regulates the transcription of genes that mediate tumour suppression. Downstream of RPL26-mediated increases in p53 translation, MDM2 acts to inhibit p53-mediated transcription and also to polyubiquitinate p53, targeting it for degradation through the proteasome. Nutlin-3 inhibits both of these effects of MDM2 on p53. (**b**) In the presence of Six1, there is a decrease in the amount of RPL26 bound to the p53 UTRs, allowing the Six1-regulated miRNA, miR-27a, to bind the p53 3′-UTR. This combined mechanism leads to a decrease in translation of p53, resulting in decreased p53-mediated transcription.

In the present study, we show that the oncoprotein Six1 is able to decrease p53 protein levels, thereby inhibiting its function. We further demonstrate that Six1 regulates p53 independent of the proteasome, and that the common regulator of p53, the E3 ubiquitin ligase, MDM2, is not required for Six1-mediated p53 downregulation. Rather, we demonstrate that Six1 decreases p53 levels via simultaneously increasing miR-27a and decreasing RPL26 (Fig. 8). Importantly, we demonstrate that Six1 is able to regulate p53 levels in several different contexts, including immortalized cells as well as cancerous cells from several tumour types, and we also demonstrate a strong correlation between Six1 and signalling downstream of p53 in cell lines and mouse models (Figs 1 and 2; Supplementary Figs 2–6). While most experiments were carried out in the presence of DNA damage to increase our ability to detect p53, it should be noted that Six1 can also decrease baseline levels of p53 in the absence of DNA damage (Figs 3a and 7h; Supplementary Fig. 6).

Further, we describe a different mechanism of regulation of p53 protein levels. We show that the sequences in the p53 3′-UTR that are necessary for the binding of RPL26 are proximal to the sequences necessary for the binding of miR-27a, and that RPL26 can compete with, and thus interfere with, the ability of miR-27a to bind to and mediate translational repression of p53 (Figs 6a and 7). While it has been previously suggested^10^,^11^, we are the first to demonstrate that RPL26 is downregulated in numerous cancers, thus underscoring a potential tumour suppressive function for the gene. This downregulation is particularly striking in breast cancer, where over 11 different microarray studies show RPL26 downregulation in breast cancer versus normal breast (Supplementary Table 1).

In addition, our studies demonstrate that Six1 upregulates miR-27a (Fig. 5d; Supplementary Fig. 15a), a previously described oncomiR, that until now was not known to target p53. miRNA-27a is overexpressed in a number of different human tumour types including gastric adenocarcinoma^32^, ovarian^55^, cervical^33^ and breast cancer^34^, all tumour types in which Six1 has been implicated^14^,^48^. Interestingly, the oncogenic properties of miR-27a have been linked to its ability to mediate an epithelial-to-mesenchymal transition^35^, to increase cell growth^32^ and to decrease apoptosis^36^, all properties that have been linked to both Six1 overexpression and p53 downregulation^16^,^23^,^48^,^56^.

While we observe a trend (*P*=0.06) towards a positive correlation between Six1 and miR-27a expression in human patient tumour data sets (Supplementary Fig. 15b), the inverse correlation between Six1 and RPL26 appears to be much stronger in patient tumours (Figs 6c–f; Supplementary Fig. 16b–h). These data imply that miR-27a may be regulated by multiple factors in addition to Six1, and that these may be context specific. In contrast, the inverse correlation between RLP26 and Six1 suggest that Six1 can regulate RPL26 in numerous contexts. Nonetheless, downregulation of RPL26 is expected to unmask the miR-27a site, and thus the mere presence of miR-27a would allow for p53 downregulation due to Six1-mediated suppression of RPL26.

Although a number of RBPs and miRNAs have been implicated in the regulation of p53 stability and expression^12^,^57^, our study is the first to highlight a competitive mechanism of action between an RBP and a miRNA on the p53 3′-UTR. Although this regulation of p53 is highly novel, several other studies have implicated competitive mechanisms of action between RBPs and miRNAs on other mRNA targets^58^,^59^,^60^. These studies, along with ours, provide a framework to describe what may turn out to be a more general mechanism of translational regulation, whereby RBPs and miRNAs can compete with each other to regulate protein translation.

Importantly, we show that the ability of Six1 to regulate p53 via a novel, MDM2-independent mechanism may have clinical consequences as Six1 overexpression induces resistance to MDM2-targeted therapies that are currently in clinical trials (Fig. 3, Supplementary Fig. 9). These drugs are efficacious in animal models, where they inhibit the p53–MDM2 interaction, thereby stabilizing p53 and allowing it to transcriptionally activate its downstream target genes to regulate cell cycle arrest, apoptosis and ultimately tumour suppression^61^. Although past studies have elegantly highlighted MDM2 as a key regulator of p53 (ref. 2), our study suggests that MDM2-targeted therapies will be less successful in tumours that have acquired other mechanisms to downregulate p53 signalling. Here we show that Six1 downregulates p53 upstream of where MDM2-targeted therapies act to stabilize it. We propose that tumours with high Six1 expression allow for less TP53 mRNA to be translated to p53 protein, such that even in the presence of MDM2-targeted therapies, not enough p53 protein is made to be stabilized by the drugs (Fig. 8). Consequently, in the presence of Six1, MDM2-targeted therapies are unable to induce adequate expression of p53 target genes, thus failing to induce apoptosis and cell cycle arrest. Given the number of different tumour types that express high levels of Six1 (refs 14, 16, 19, 24, 47, 48, 62), it may be important to assess Six1 levels before carrying out clinical trials with p53–MDM2 interaction inhibitors. In addition, gaining an understanding of how Six1 regulates miR-27a and RPL26 may be important in the future for identifying novel ways to resensitize tumours to Nutlin-3 therapies.

## Methods

### Array analysis

For the hierarchical clustering gene expression analysis of GEMMs that correspond to different breast cancer subtypes, 377 Agilent mouse tumour microarrays were utilized^20^. Data from these arrays were downloaded and probes with 70% successful binding (binding of both the experimental RNA sample and the reference RNA channel for each probe) were used. Using R Project software^63^ version 3.1, missing array values were imputed with the *k*-Nearest Neighbour algorithm (*k*=10). A microarray platform correction as described^20^ was then applied to eliminate any bias due to chip effects. Cluster version 3.0 was then used to median centre the genes across all 377 arrays, and hierarchical clustering of genes and arrays was performed with Similarity Metric ‘Correlation (uncentred)' using the Centroid Linkage Clustering method. Microarray analyses for 66Cl4 scrambled and Six1 KD cells were performed using the Affymetrix MoGene 1.1 ST GeneChip in triplicate at the Genomics and Microarray Shared Resource of the University of Colorado Cancer Center. Gene expression profiles were extracted and normalized with a robust multiarray average (RMA) using Affymetrix Power Tools. Gene Set Enrichment Analysis was performed on the scrambled versus the Six1 KD cells with the mouse gene sets from the Kyoto Encyclopedia of Genes and Genomes (mmu, version september 2010). Gene sets were permutated 1,000 times and gene sets that have *P*<0.05 were considered significant. All microarray data have been deposited in the NCBI GEO database (accession numbers GSM1589824 and GSM1589833).

### HITS-CLIP

HITS-CLIP on MCF7 and BT-474 cells was performed previously^37^ according to Chi *et al*.^64^ protocol, with minor modifications. For full protocol, see ref. 65. Briefly, ∼15–20 × 10^6^ cells were cross-linked with 600 mJ cm^−2^ of short-wave ultraviolet radiation (254 nm) in split doses. Cells were lysed and argonaute proteins 1–4 (AGO1–4), along with associated cross-linked RNA, were IP with a murine monoclonal antibody (2A8 (32); gift from Dr Zissimos Mourelatos (University of Pennsylvania, Philadelphia, PA). A ^32^P-labelled 3′-RNA linker was ligated to the RNA in the IP Ago–RNA complexes ‘on-bead.' The Ago–RNA complexes were resolved by SDS–polyacrylamide gel electrophoresis, transferred to nitrocellulose and visualized by autoradiography to determine localization of the Ago–RNA complexes. These complexes were excised, RNA extracted and reverse transcribed to complementary DNA (cDNA). A cDNA library suitable for sequencing on the Illumina platform was then constructed with serial amplification by PCR. Sequencing was performed on an Illumina Genome Analyzer IIx (single end 50 base pair reads).

### Cell culture

The generation of MCF7-Ctrl and Six1 (ref. 62) lines, MCF12A-Ctrl and Six1 (ref. 21), and 66Cl4 Six1 KD lines^15^ was previously described. For clonal isolates of MCF7 and MCF12A cell lines, each experiment shown displays one Six1 and one Ctrl clone due to space constraints, but each experiment is representative of data seen across at least two sets of clones. Transient gene KD experiments were performed using ON-TARGETplus SMARTpool siRNA constructs (Supplementary Table 2) against the gene of interest. Transient overexpression of Six1 was performed using a pcDNA3.1 vector containing full-length Six1 (ref. 47). eGFP-RPL26 and pGL3ctrl luciferase vector flanked by TP53 UTRs were gifts from Dr Kastan (Addgene plasmid #31980 and #28175). Mutations were introduced by site-directed mutagenesis with the use of the Quickchange II kit (Agilent Technologies). Transient overexpression of miRNAs was performed using miRIDIAN mimics (Dharmacon). All cell lines were profiled to confirm their identity and periodically checked for mycoplasma contamination. For cell culture conditions and drug additions, see Supplementary Tables 3 and 4.

### Immunocytochemistry

Cells (100,000) were plated on coverslips. After 48 h, cells were treated with etoposide and then 24 h later fixed in 4% paraformaldehyde and permeabilized with 0.2% TritonX100 in PBS. After blocking in 2% goat serum, cells were incubated in specific primary followed by secondary antibodies for 30 min at room temperature. Coverslips with cells were then mounted onto glass slides using vecta-shield containing 1 μg ml^−1^ 4,6-diamidino-2-phenylindole (DAPI) to stain the nuclei.

### ChIP–qPCR

Cells were treated with etoposide (10 μM) or DMSO. After 4 h of treatment at 37 °C, cells were cross-linked with a 1% formaldehyde/PBS solution for 15 min at room temperature. Cross-linking was stopped by the addition of 125 mM glycine, cells were washed with cold PBS and then collected in RIPA buffer (150 mM NaCl, 1% v/v Nonidet P-40, 0.5% w/v deoxycholate, 0.1% w/v SDS, 50 mM Tris pH 8, 5 mM EDTA, 20 mM NaF, 0.2 mM sodium orthovanadate, 5 μM trichostatin A, 5 mM sodium butyrate and protease inhibitors). Samples were sonicated to generate <500-bp fragments. For immunoprecipitation, 1 mg of protein extract was first pre-cleared with 30 μl of 50% Protein G sepharose (GE Healthcare) for 2 h at 4 °C. 0.5 μg of antibody was added (p53 Calbiochem OP43, and Normal Mouse IgG SC-2025) to appropriate samples and incubated overnight at 4 °C with 30 μl of protein-G beads pre-blocked with 1 mg ml^−1^ bovine serum albumin and 0.3 mg ml^−1^ salmon sperm DNA. Beads were then washed twice with RIPA, four times with ChIP bead wash (100 mM TrisHCL pH 8.5, 500 mM LiCL, 1% v/v Nonidet P-40, 1% w/v deoxycholic acid), twice more with RIPA and then twice with cold 1 × TE. Immunocomplexes were eluted with 1% SDS for 10 min at 65 °C, and cross-link reversed by incubating for 5 h at 65 °C. DNA was purified and a fraction was used as a template in real-time PCR with primers towards the p21 distal (−2,283) and proximal (−1,391) p53-binding sites, as well as a non-p53-binding site (+11,443) ∼1,200-bp away. qPCR values were normalized to input values for each cell line, which were extracted from 100 μg protein extract.

### UTR luciferase assays

Cells were plated to equal confluence and indicated luciferase constructs were co-transfected along with a renilla luciferase construct under the control of a constitutively active CMV promoter to control for transfection efficiency. At 24–48 h after transfection, cells were collected and analysed for luminescence using the Dual-luciferase Reporter Assay System (Promega). To normalize experiments, parallel wells were transfected with an empty vector luciferase control construct and a renilla luciferase construct. Empty vector control values were then subtracted from the corresponding experimental values.

### Western blot analyses

Whole-cell extracts and nuclear extracts were isolated as previously described^16^. Proteins were electrophoresed and then transferred to polyvinylidene difluoride membranes. After blocking in 5% milk in Tris-buffered saline with 0.1% tween 20, membranes were incubated in specific antibodies overnight at 4 °C (see Supplementary Table 5 for specific antibodies). For Six1 protein detection, antibodies from Sigma (HPA001893) and antibodies generated in our laboratory as previously described^66^ were used interchangeably. Image lab software was used to quantify western blot analysis. Uncropped images are shown in Supplementary Fig. 18.

### qRT–PCR

Total RNA was extracted using the RNAeasy or miRNeasy RNA isolation kit (Qiagen). cDNA synthesis was performed using iScript (Biorad) for mRNA qRT–PCR and miScript (Biorad) for miRNA qRT–PCR. All mRNA qRT–PCR assays were performed using ssoFast Evagreen supermix (BioRad) (primer sequences are shown in Supplementary Table 6). miR-27a assays were performed using miScript primer assay (Qiagen MS00003241). Assays were performed using the Biorad CFX96.

### Colony formation assay

A total of 300 cells per well (in a six-well plate) were plated in triplicate. Cells were treated for 48 h with Nutlin-3 or DMSO at indicated concentrations, washed with fresh media and allowed to grow to form single-cell-derived colonies. Five days later, colonies were stained with crystal violet (0.1% in 25% methanol in H_2_O), counted (if they were 50 cells or greater) and normalized to the number of colonies found in the corresponding DMSO-treated samples for each cell line.

### Bromodeoxyuridine and flow cytometry

After indicated treatments, cells were incubated for 1 h with BrdU (10 μM), and were then trypsinized. Cells were fixed, then incubated with anti-mouse IgG-FITC, after which they were stained with propidium iodide. Analysis was performed using the Beckman Coulter FC500 flow cytometer.

### xCELLigence

Cells were plated to equal confluence on E-plates in triplicate (ACEA biosciences Inc.). At 24 h after plating, cells were treated with indicated drug doses and analysed on the xCELLigence RTCA MP system with impedance readings taken every 30 min to 4 h. Cells were retreated with drugs every 48–72 h and impedance measurements were taken to calculate the relative quantity of confluence for attached cells to the bottom of each well.

### MTS assay

Cells were plated to equal confluence in 96-well plates in triplicate and treated with indicated dose of Nutlin-3. After indicated incubation times, the 3-(4,5-dimethylthiazol-2-yl)-5-(3-carboxymethoxyphenyl)-2-(4-sulfophenyl)-2H-tetrazolium and phenazine methosulfate (MTS) reagents were added to each well as per the manufacturer's instructions (Promega). Plates were incubated at 37 °C for 2–4 h and absorbance was measured 490 nm.

### Patient-derived xenografts

Frozen tissue was used from previously described PDXs^67^ (The Breast Cancer Tissue Bank IRB # 04-0066. Approval date is 9 November 2014. Patient consent was obtained) in which a fragment of flash-frozen tumour was pulverized with a tissue pulverizer in liquid nitrogen. Approximately 100–200 mg of tissue was lysed with RIPA buffer plus protease inhibitors. Protein was determined via BIORAD assay followed by western blot analysis.

### Statistical analysis

When exactly two conditions were compared, an unpaired one-tailed Student's *T*-test was used, abbreviated in the figure legends as ‘*T*-test'. When two or more conditions were compared, a one-way analysis of variance followed by a Tukey's multiple comparison test was used, abbreviated in the figure legends as ‘analysis of variance'. Specific statistical analyses used for each array is described accordingly in each figure legend.

## Additional information

**Accession codes:** The microarray data for the GEMMs and 66Cl4 Six1 KD cell lines have been deposited in the Gene Expression Omnibus under accession codes GSM1589824, GSM1589833 and GSE65200 respectively.

**How to cite this article:** Towers, C. G. *et al*. The Six1 oncoprotein downregulates p53 via concomitant regulation of RPL26 and microRNA-27a-3p. *Nat. Commun.* 6:10077 doi: 10.1038/ncomms10077 (2015).

## Supplementary Material

Supplementary Information Supplementary Figures 1-18 and Supplementary Tables 1-6
