# Supplementary material for: The Six1 oncoprotein downregulates p53 via concomitant regulation of RPL26 and microRNA-27a-3p
Source: Nat Commun. 2015 Dec 21;6:10077. doi: 10.1038/ncomms10077 (PMC4703841; doi:10.1038/ncomms10077)
Supplement: Supplementary Information — Supplementary Figures 1-18 and Supplementary Tables 1-6 [file ncomms10077-s1.pdf]

Supplementary Figure-1 (Ford)

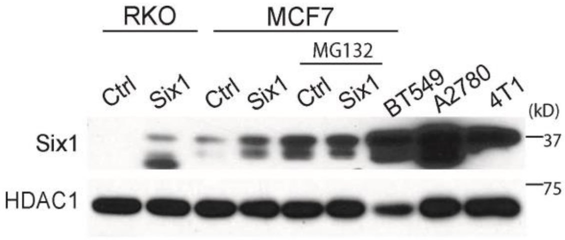

Supplementary Figure 1: Ectopic Six1 over expression is within a physiologically relevant range. Western blot analysis performed on nuclear lysates (for RKO, MCF7, BT549 and A2780 cell) and whole cell lysates (for 4T1 cells) demonstratingthat stable overexpression of Six1 does not exceed endogenous Six1 expression in multiple cancer cell lines.

Supplementary Figure-2 (Ford)

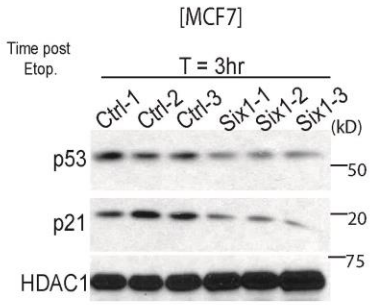

Supplementary Figure 2: Six1 decreases p53 in MCF7 cells. Western blot analysis performed on nuclear lysates from MCF7-Six1 and Ctrl clonal isolates 3hrs post etoposide treatement (10μM) shows a decrease in p53 and p21 protein expression in all three clones examined.

Supplementary Figure-3 (Ford)

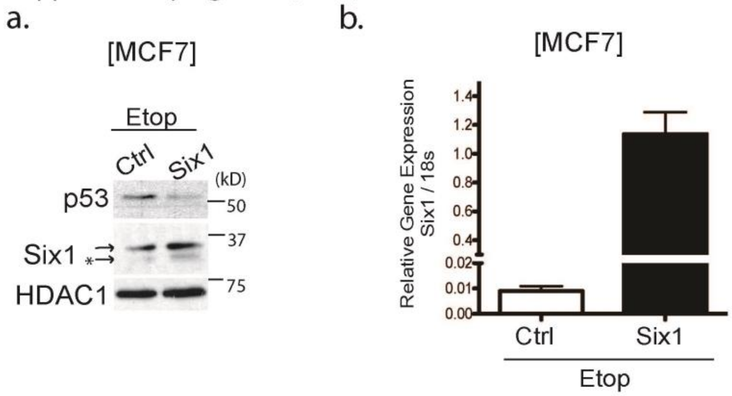

Supplementary Figure 3: Six1 overexpression is maintained in the context of DNA damage (a) Western blot analyses in MCF7-Ctrl and Six1 cell lines 3hrs post etoposide (10μM). (b) qRT-PCR in MCF7-Ctrl and Six1 cell lines 3hrs post etoposide (10μM). Mean±s.d of replicates of 3 for a representative experiment (2 experiments in total).
.

Supplementary Figure-4 (Ford)

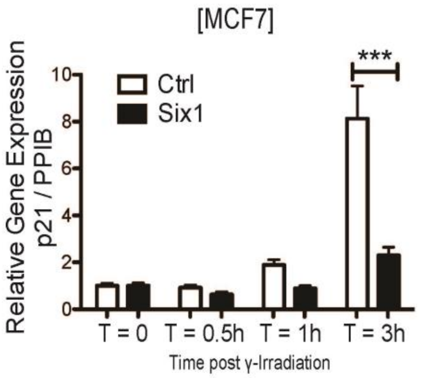

Supplementary Figure 4: Six1 decreases p21 mRNA. qRT-PCR in MCF7-Ctrl and Six1 overexpressing cells shows a decrease in γ-irradiation induced p21 mRNA expression at multiple time points post γ-irradiation treatement (20Gy). Gene expression is normalized to PPIB. ANOVA on mean±s.d of triplicate samples for a representative experiment (of 2 experiments). *** $p < 0.001$

Supplementary Figure-5 (Ford)

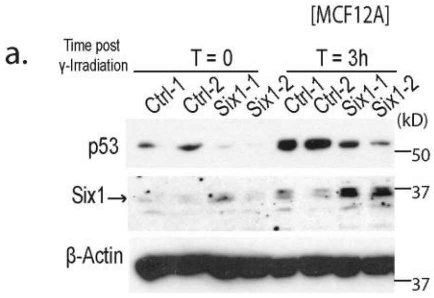

Supplementary Figure 5: Six1 decreases p53 in MCF12A cells. Western blot analysis performed on whole cell lysates harvested after 5Gy of X-irradiation for indicated time points from MCF12A cells stably transfected with a control (Ctrl) or Six1-expressing vector demonstrates a decrease in p53 protein expression.

Supplementary Figure-6 (Ford)

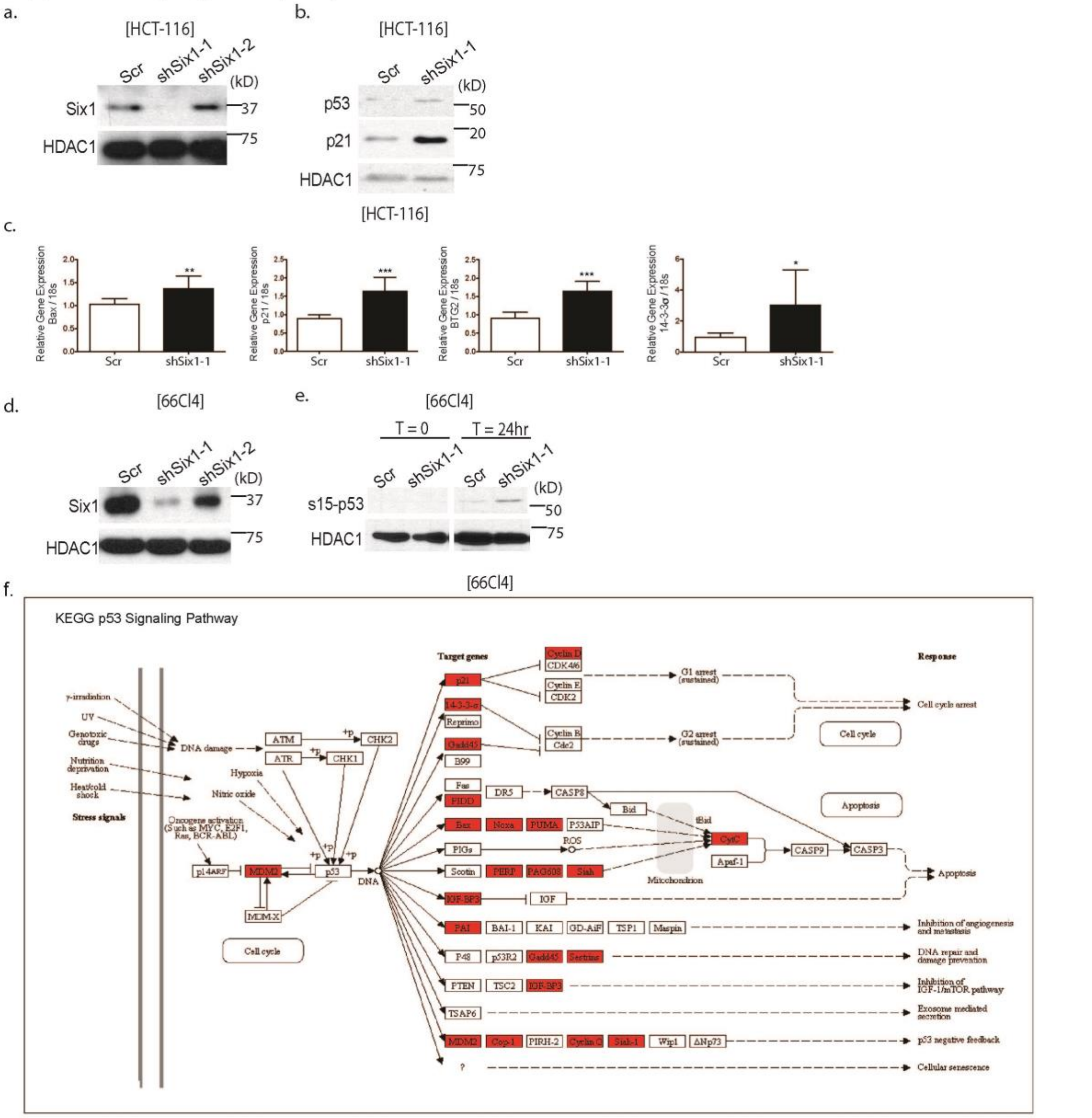

Supplementary Figure 6: Knockdown of Six1 causes an increase in p53 and downstream signaling (a) Western blot analysis on nuclear lysates from HCT-116 cells showing stable knockdown of Six1with one knockdown construct. (b) Western blot analysis on nuclear lysates from HCT-116 shSix1-1 shows an increase in p53 and p21 protein expression. (c) qRT-PCR from HCT-116 shSix1-1 cells shows an increase in p53 target gene expression. Student's T-test on mean±s.d of 3 combined experiments (total n=9). (d) Western blot analysis on nuclear lysates from 66Cl4 cells showing stable knockdown of Six1 with one knockdown construct. (e) Western blot analysis on nuclear lysates from 66Cl4 shSix1-1 cells reveals an increase in s15-phosphorylated p53 protein expression 24hrs post etoposide (10μM). Blots were cropped to remove unnecessary lanes. (f) Microarray analysis was performed on mRNA from 66Cl4 stable Six1 knockdown cells and pathway analysis utilizing the KEGG pathway for p53 was then performed. Genes in red were significantly upregulated in shSix1-1 knockdown cells as compared to the scramble control.

Supplementary Figure-7 (Ford)

a.

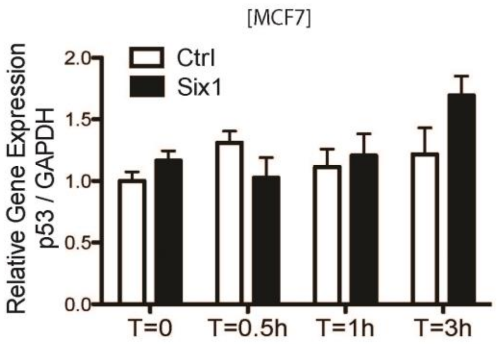

b.

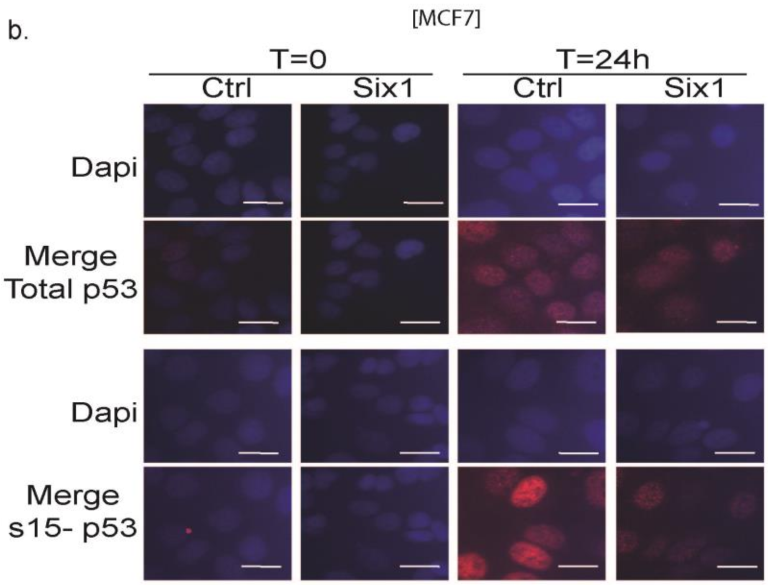

Supplementary Figure 7: Six1 does not affect p53 mRNA levels nor nuclear localization of the p53 protein
(a) Stable overexpression of Six1 in MCF7 cells does not decrease p53 mRNA expression before or up to 3 hours after 20Gy of γ-irradiation. The data is shown as the mean±s.d of triplicate samples for a representative experiment (of 2 experiments) (b) Immunocytochemistry performed 24hrs after etoposide treatement (10μM) demonstrates that stable overexpression of Six1 in MCF7 cells causes a decrease in total p53 and s15-phospho-p53, but does not alter localization of p53. Scale bar = 50μM, original magnification X400.

Supplementary Figure-8 (Ford)

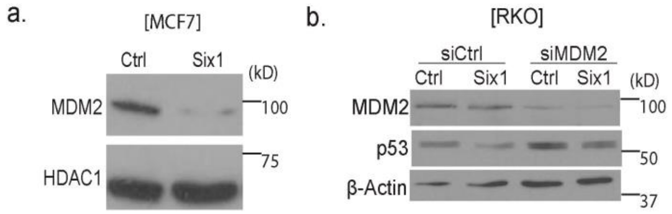

Supplementary Figure 8: Six1 does not upregulate MDM2 in order to regulate p53. (a) Western blot analysis performed on whole cell extracts demonstrates that Six1 overexpression in MCF7 cells causes a decrease in MDM2. (b) Western blot analysis performed in whole cell extracts from RKO-Ctrl and Six1 cells 24hrs after transient transfection with an siRNA pool against MDM2 shows that Six1 maintains its ability to decrease p53 with knockdown of MDM2.

Supplementary Figure-9 (Ford)

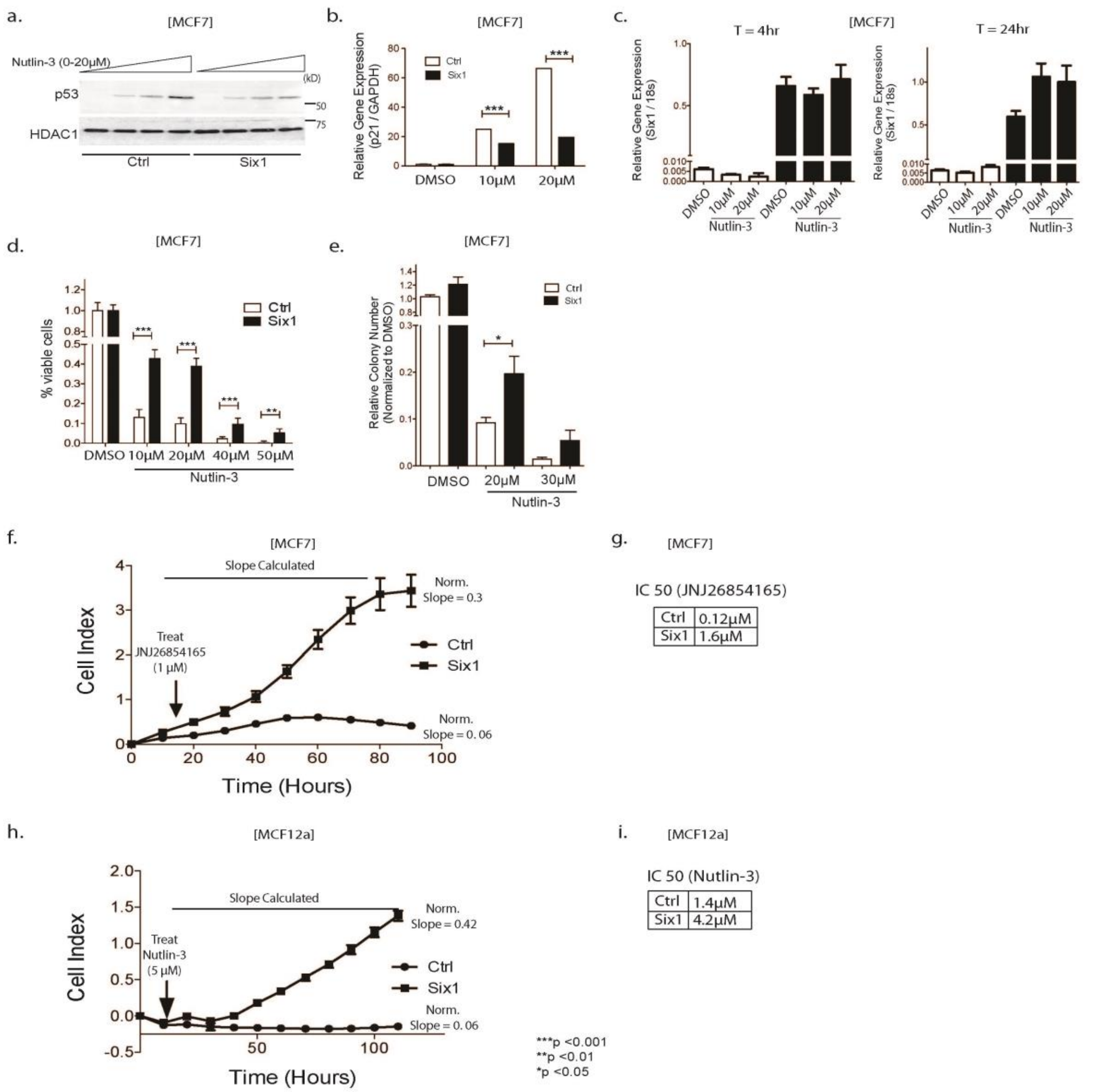

Supplementary Figure 9: Six1 downregulates p53 via an MDM2 independent mechanism, inducing resistance to MDM2 targeted therapies. (a) Western blot analysis performed on nuclear lysates from MCF7-Ctrl and Six1 cell lines 24hrs post treatment with Nutlin-3 (b) qRT-PCR for p21 in MCF7-Ctrl and Six1 cell lines 24hrs post treatment with Nutlin-3. Gene expression is normalized to GAPDH. ANOVA on Mean±s.d for triplicate samples for representative experiment (of 3 experiments). (c) qRT-PCR for Six1 in MCF7-Ctrl and Six1 cell lines 4hr and 24hrs post treatment with Nutlin-3. Mean±s.d for triplicate samples. (d) MTS assay performed 96hrs after cells were treated with Nutlin-3. ANOVA on mean±s.d of biological replicates of 6 for a representative experiment (of 3 experiments). (e) Colony formation assay in MCF7-Ctrl and Six1 cell lines treated with Nutlin-3. Colony number was normalized to the DMSO treated sample for corresponding cell lines. Mean±s.d of triplicate samples from 2 combined experiments (total n= 6). (f) An xCELLigence cell proliferation assay performed on MCF7-Six1 and Ctrl cells plated in triplicate to equal confluence. Impedance was measured every 30 minutes and demonstrates that Six1 inhibits the decrease in cell growth caused by treatment with JNJ26854165 (Serdementan) for 90hrs. Representative figure of two experiments shown. The slope of the growth curves was calculated after treatment with JNJ26854165, and normalized to the slope of the growth curves after treatment with DMSO. The data shown is a representative figure (of 2 experiments) and is shown as the mean±s.d. of triplicate samples. (g) IC50s calculated from the growth curves generated with MCF7-Ctrl and Six1 cell lines treated across a range of doses of JNJ26854165 (0-40μM). (h) An xCELLigence cell proliferation assay performed on MCF12A-Six1 and Ctrl cells plated in triplicate to equal confluence. Impedance was measured every 30 minutes. The slope of the growth curves was calculated after treatment with Nutlin-3, and normalized to the slope of the growth curves after treatment with DMSO. The data shown is a representative figure (of 2 experiments) and is shown as the mean±s.d. of triplicate samples. (h) IC50s calculated from the growth curves generated with the MCF12A-Ctrl and Six1 cell lines treated across a range of doses of Nutlin-3 (0-40μM).

Supplementary Figure-10 (Ford)

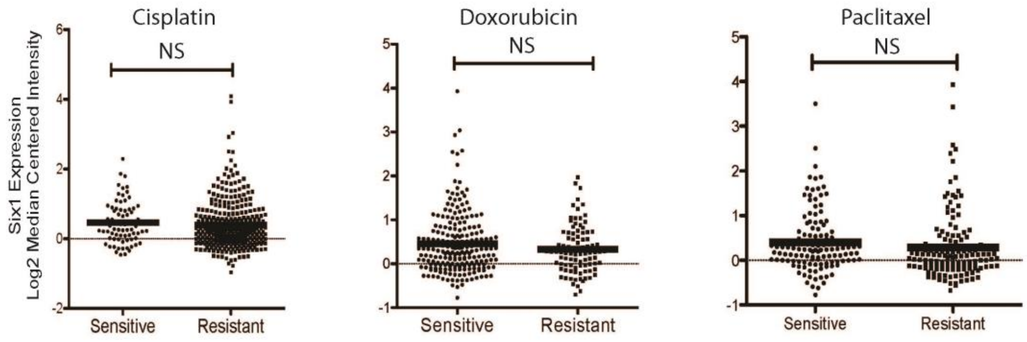

Supplementary Figure 10: Six1 does not cause resistance to general chemotherapies. Examination of a publicly available dataset of cancer cell lines representing multiple tumor types shows that overexpression of Six1 does not correlate with resistance to cisplatin, doxorubicin or paclitaxel.

Supplementary Figure-11 (Ford)

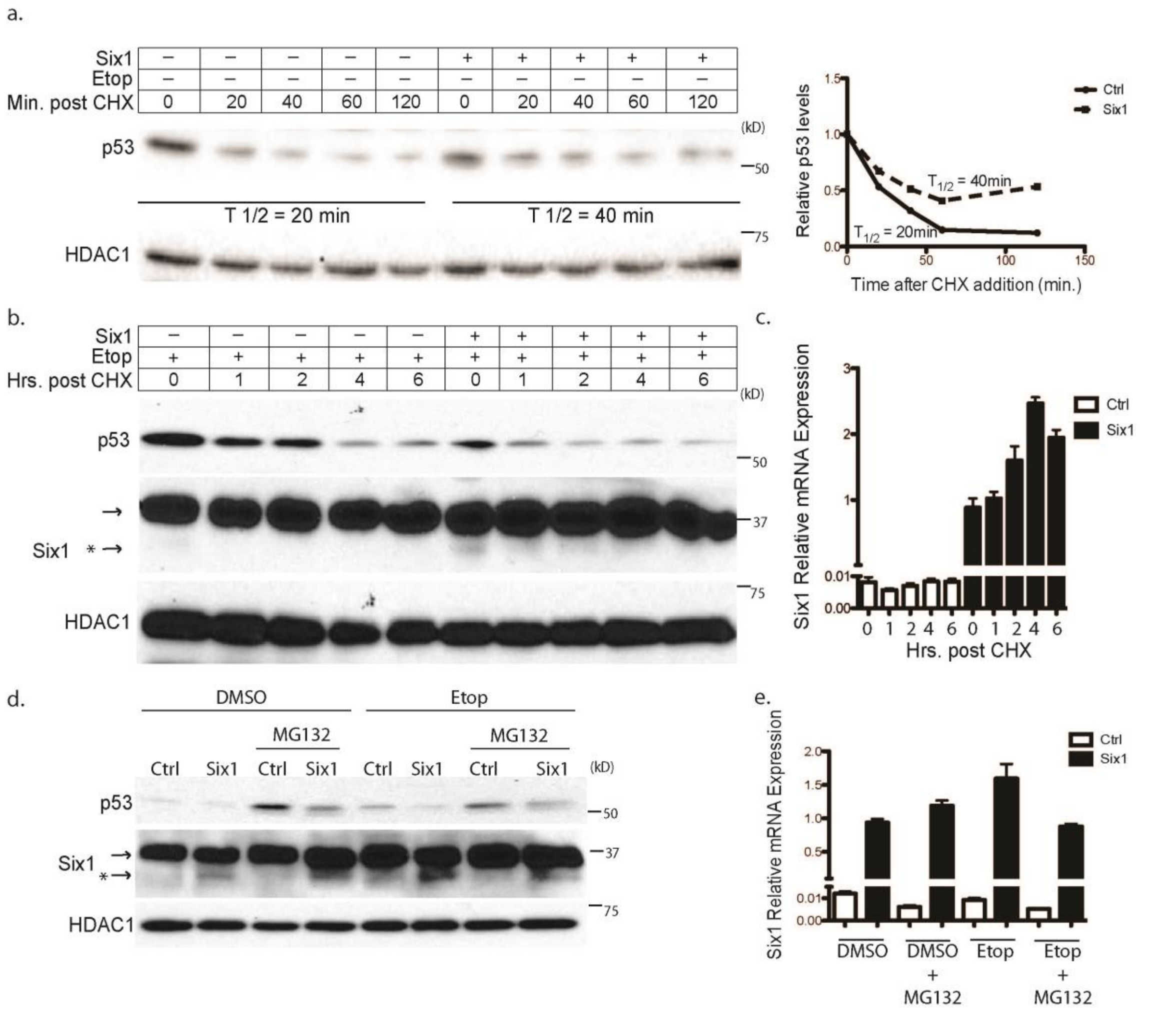

Supplementary Figure 11: Six1 does not decrease the half-life of p53. (a) Western blot analysis from nuclear lysates from MCF7-Ctrl and Six1 cells treated with cycloheximide (20μM) and harvested after indicated periods of time shows no decrease in the p53 half life in the presence of Six1 overexpression and absence of DNA damage. Right: p53 half-life was calculated after quantifying signal on Western blots using Image Lab software. (b) Western blot analysis from nuclear lysates from MCF7-Ctrl and Six1 cell lines shows that overexpression of Six1 is maintained in the presence of cycloheximide (20μM) and etoposide (10μM) Note: the top band is a phosphorylated and inactive form of Six1 and the lower band denoted by * is the active form of Six1. (c) qRT-PCR from MCF7-Ctrl and Six1 cell lines confirms that overexpression of Six1 is maintained in the presence of cycloheximide (20μM) and etoposide (10μM). (d) Western blot analysis performed on nuclear lysates from MCF7-Ctrl and Six1 cells shows that Six1 overexpression is maintained in the presence of MG132 (25μM) and etoposide (10μM). (e) qRT-PCR from MCF7-Ctrl and Six1 cell lines confirms that overexpression of Six1 is maintained in the presence of MG132 (25μM).

Supplementary Figure-12 (Ford)

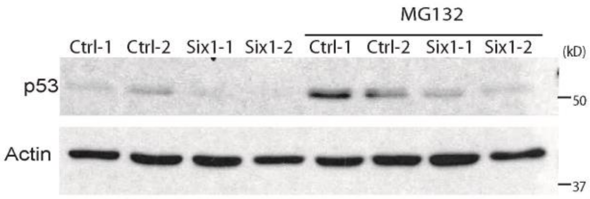

Supplementary Figure 12: Inhibition of the proteosome does not rescue Six1 mediated regulation of Six1 in the MCF12A cells: Western blot analysis performed on cell lysates from MCF12A-Ctrl and Six1 cell lines treated with MG132 (25μM).

Supplementary Figure-13 (Ford)

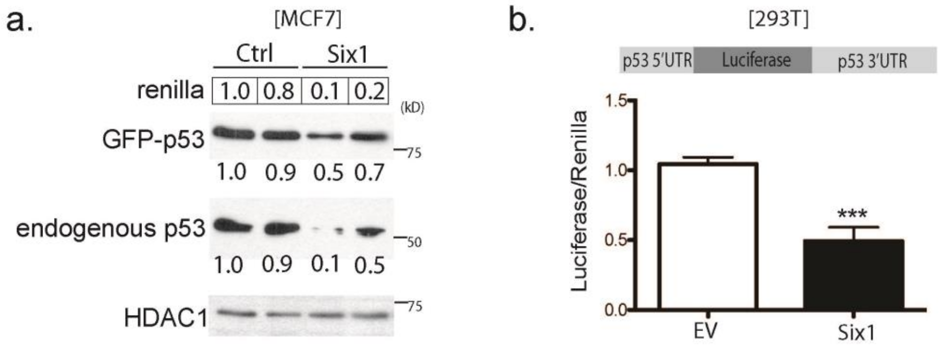

Supplementary Figure 13: The UTR regions are neccessary and sufficient for Six1 mediated regulation of p53. (a) Western blot analysis performed on nuclear lysates from MCF7-Ctrl and Six1 cells that were co-transfected with GFP-p53 and a constitutively active renilla-luciferase construct. After 1hr of etoposide treatment (10μM), cell pellets were divided to obtain nuclear lysates and perform renilla luciferase assays to determine transfection efficiency.
(b) Luciferase assay performed in 293T cells transfected with a firefly luciferase vector containing 145 base pairs of the p53 5'UTR and the entire p53 3'UTR along with a renilla luciferase vector (as a control for transfection efficiency), as well as transient Six1n (or empty vector). The data is shown as the mean±s.d of a representative experiment (of 3 experiments) run in biological triplicate and P values were calculated using an unpaired two-tailed students T-test.

Supplementary Figure-14 (Ford)

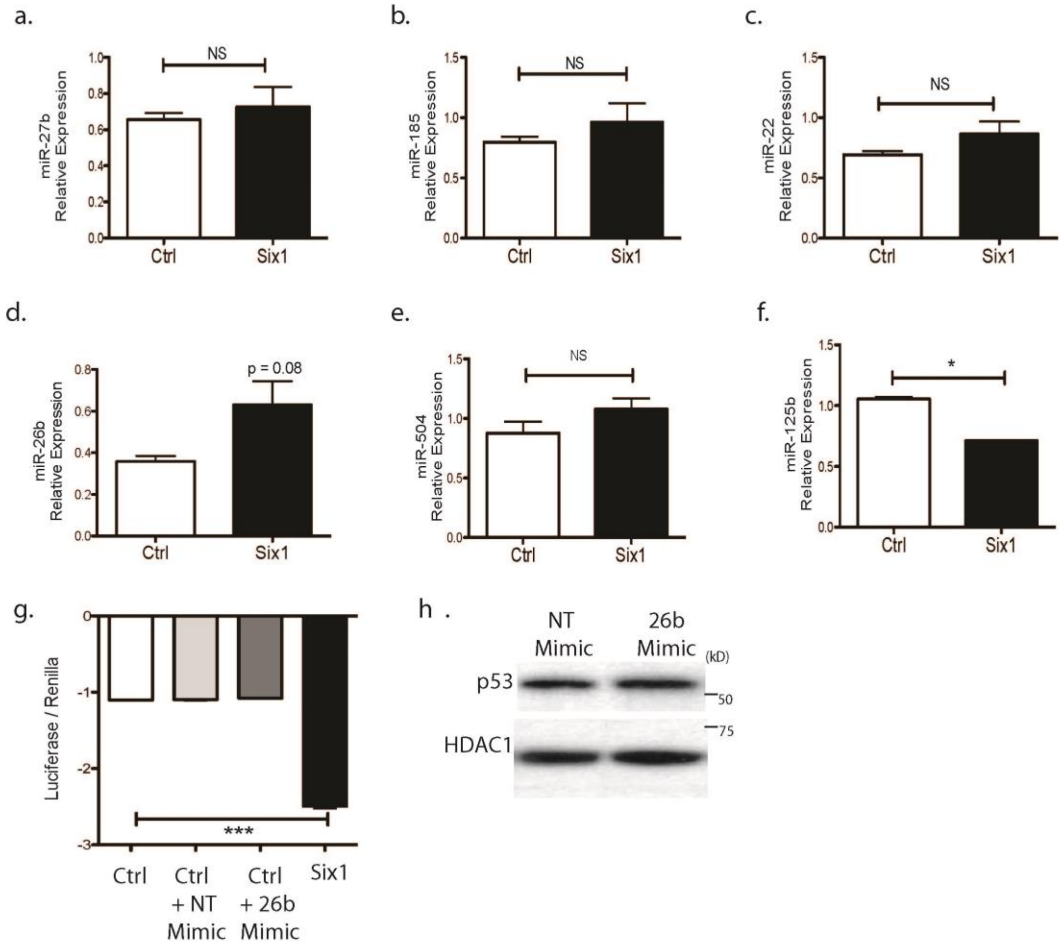

Supplementary Figure 14: Six1 does not regulate other microRNAs predicted to target p53. (a-d) qRT-PCR performed on RNA isolated from MCF7-Ctrl and Six1 cells for microRNAs identified as putative targets of Six1 on a microRNA microarray that are predicted to bind the p53 3'UTR. Data shown are the mean±s.d of triplicate samples. (e-f) qRT-PCR in MCF7-Ctrl and Six1 cells for microRNAs shown in the literature to bind the p53 3'UTR. Data shown are the mean±s.d of triplicate samples. (g) Luciferase assay performed in MCF7-Ctrl and Six1 cells transfected with a NT mimic or miR-26b mimic along with a firefly luciferase vector containing the entire p53 3'UTR and a renilla luciferase vector (as a control for transfection efficiency). ANOVA performed on the mean±s.d of a triplicate samples. (h) Western blot analysis for p53 performed on nuclear lysates from MCF7 cells transiently transfected with a NT mimic or miR-26b mimic.

Supplementary Figure-15 (Ford)

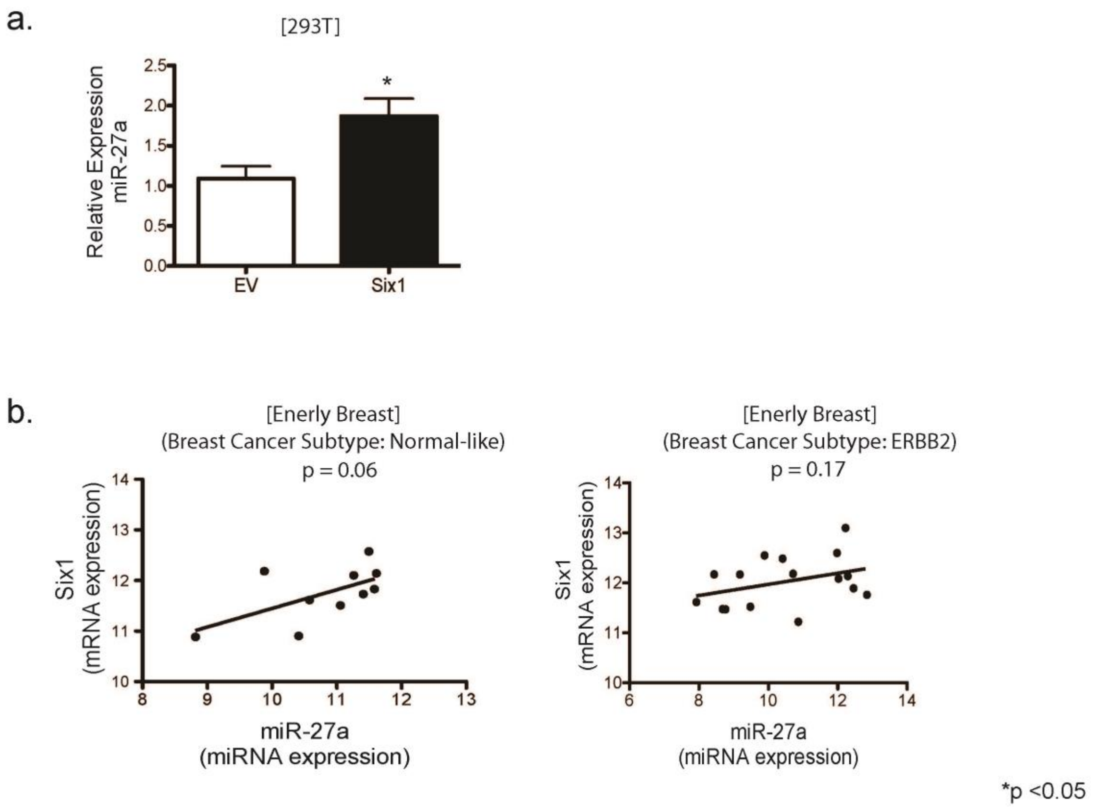

Supplementary Figure 15: Six1 regulates miR-27a. (a) qRT-PCR confirms that transient overexpression of Six1 upregulates mature miR-27a expression in 293T cells. The data is shown as the mean±s.d of triplicate samples of a representative experiment (of 2 experiments) and p values were calculated using an unpaired two-tailed students T-test. (b)Linear regression analyses examining the positive correlation between Six1 and miR-27a expression in patient tumors obtained from the Enerly Breast cancer data set.

Supplementary Figure-16 (Ford)

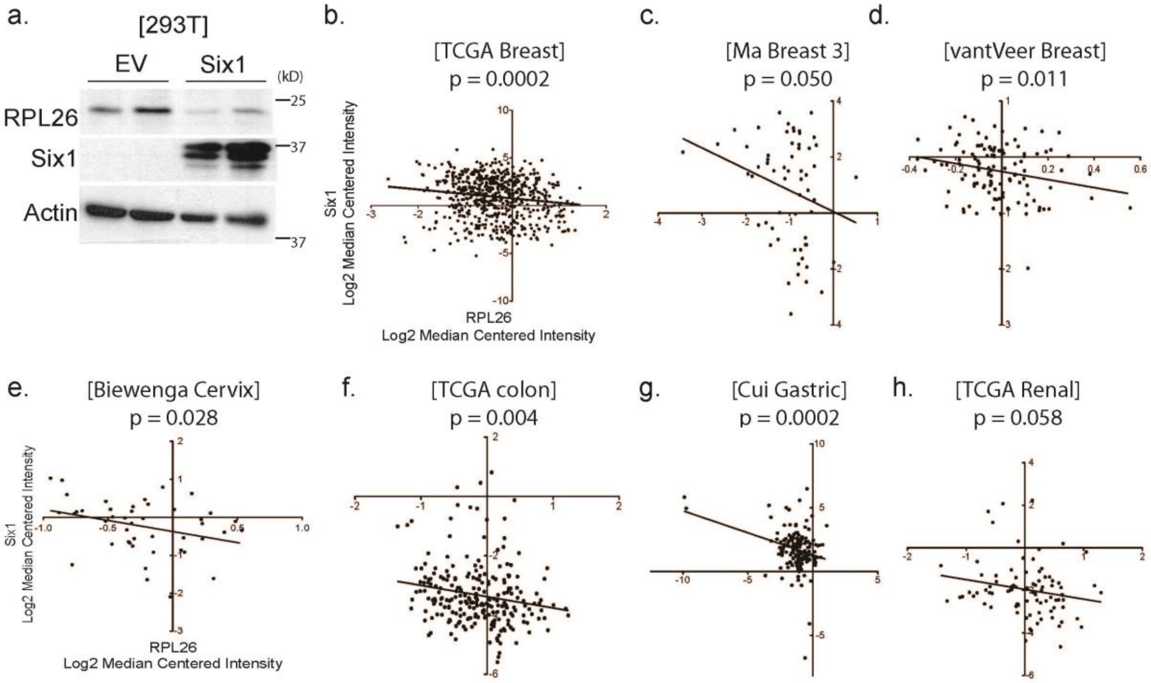

Supplementary Figure 16: Six1 decreases RPL26 expression. (a) Western blot analysis performed on whole cell lysates harvested from 293T cells with transient overexpression of Six1 or an empty control vector shows that Six1 decreases RPL26 protein levels. Samples were electrophoresed in biological duplicates. (b-h) Linear regression analyses examining the inverse correlation between Six1 and RPL26 expression in patient tumors obtained from the (b)TCGA Breast, (c) Ma Breast 3, (d) vantVeer Breast, (e) Biewenga Cervix, (f) TCGA Colon, (g) Cui Gastric, and (h) TCGA Renal cancer data sets via Oncomine.

Supplementary Figure-17 (Ford)

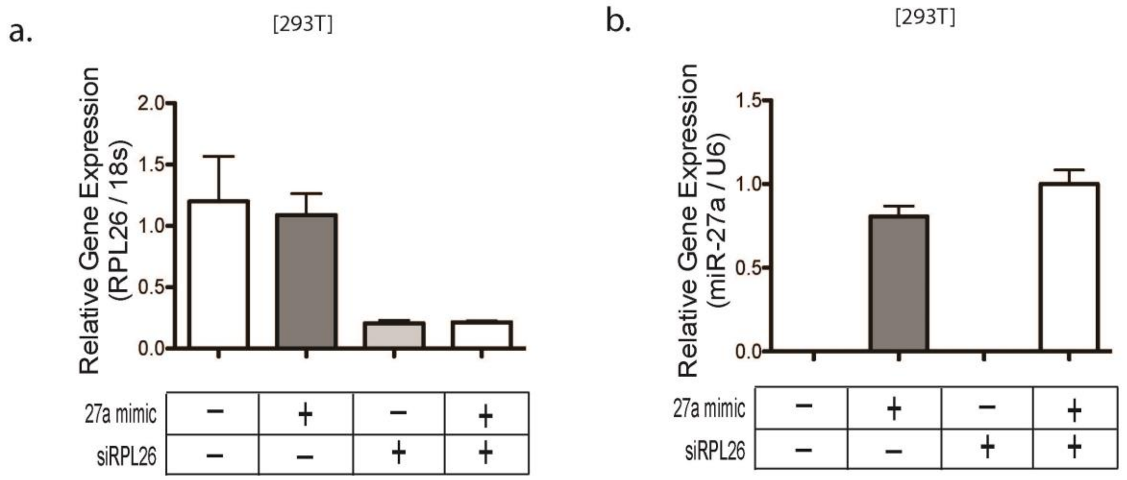

Supplementary Figure 17: Confirmation that transient transfection of siRPL26 causes knockdown of RPL26 and that transient overexpression of miR-27a mimic leads to increased levels of miR-27a. qRT-PCR in 293T cells shows (a) a decrease in RPL26 expression 48hrs after transient transfection with a pool of siRNAs against RPL26. The data is shown as the mean±s.d of triplicate samples and is representative of all transfections performed with siRPL26. (b) an increase in mature miR-27a expression 48hrs after transient transfection with miR-27a mimic. The data is shown as the mean±s.d of triplicate samples and is representative of all transfections performed with miR-27a mimic. Note: when (–) are used, the correct non-targeting control was transfected into the cells.

Supplementary Figure-18 (Ford)

Figure 2a (p53)

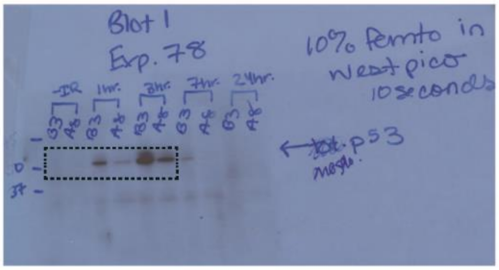

Figure 2a (p21)

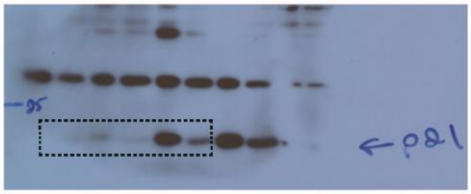

Figure 2a (HDAC1)

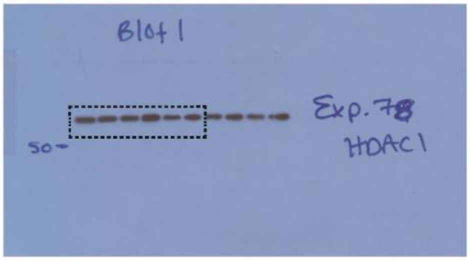

Figure 2a (HDAC1)

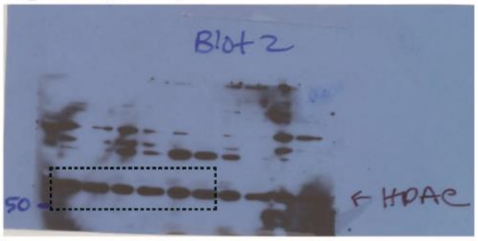

Figure 2b (p53)

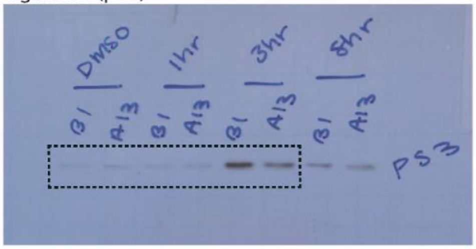

Figure 2b (p21)

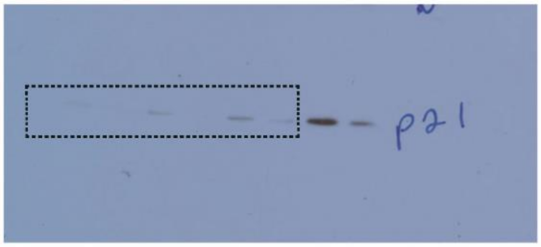

Figure 2b (HDAC1)

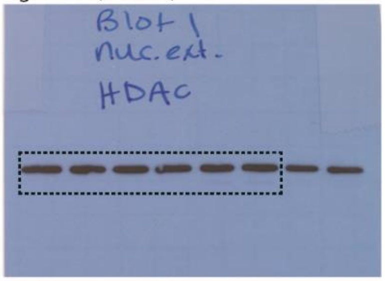

Supplementary Figure-18 (Ford)

Figure 2e (p53)

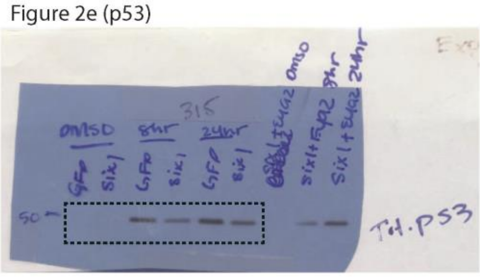

Figure 2e (Six1)

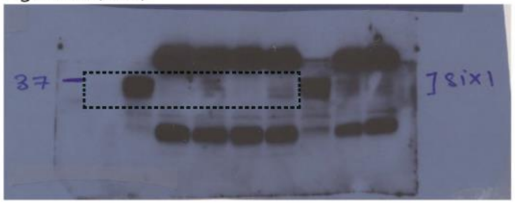

Figure 2f (p53)

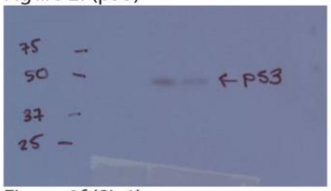

Figure 2f (Six1)

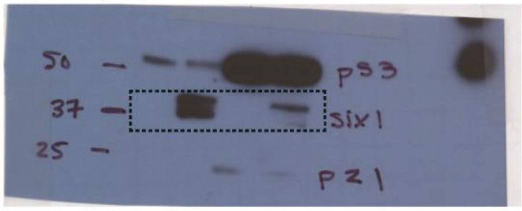

Figure 2g (p53)

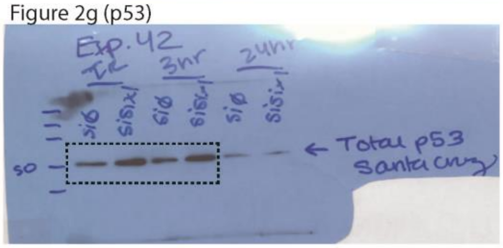

Figure 2g(Six1)

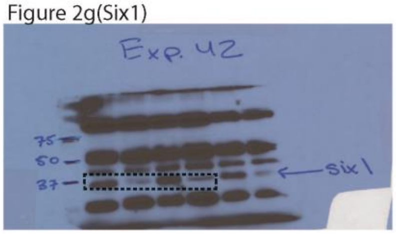

Figure 2e (p21)

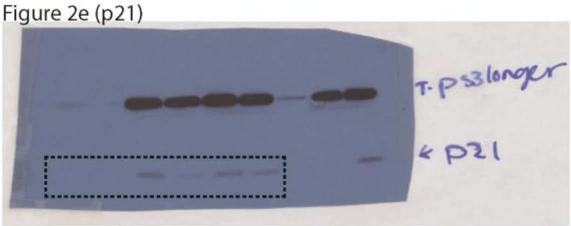

Figure 2e (HDAC1)

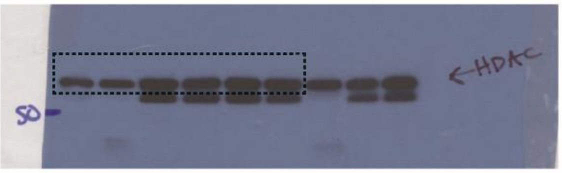

Figure 2f (p21)

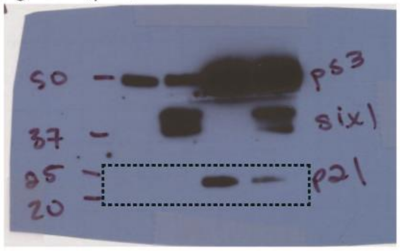

Figure 2f (HDAC1)

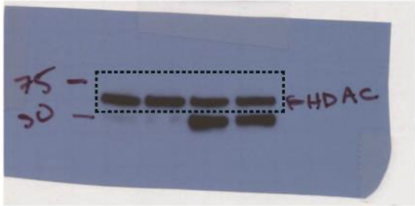

Figure 2g(s15-p53)

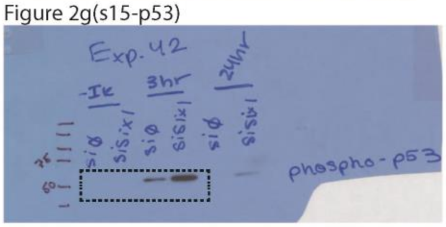

Figure 2g (β-Actin)

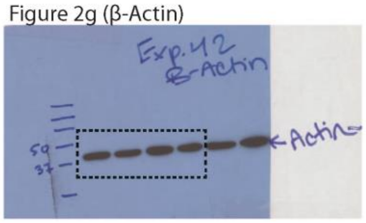

Supplementary Figure-18 (Ford)

Figure 3a (p53)

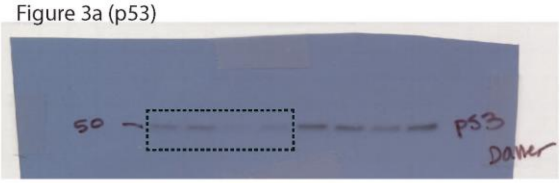

Figure 3a (HDAC1)

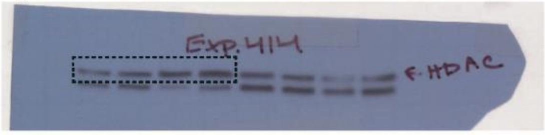

Figure 3a (cleaved parp)

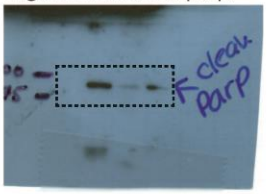

Figure 3a (β-Actin)

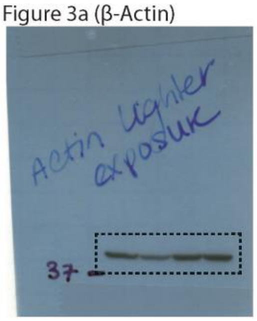

Figure 3a (MDM2 - NE)

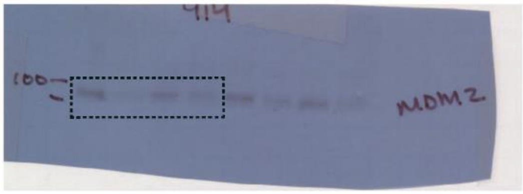

Figure 3a (MDM2-CE)

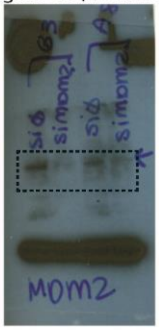

Figure 3a (p21)

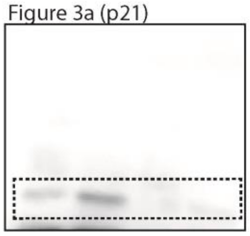

Figure 3c (p53)

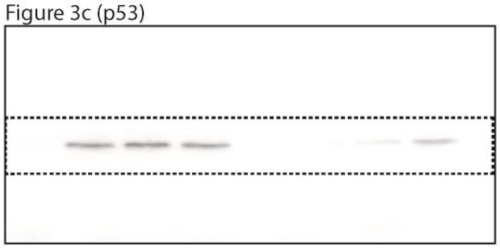

Figure 3c (p21)

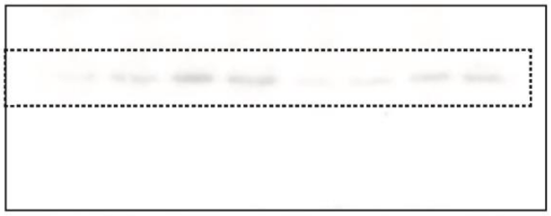

Figure 3c (HDAC1)

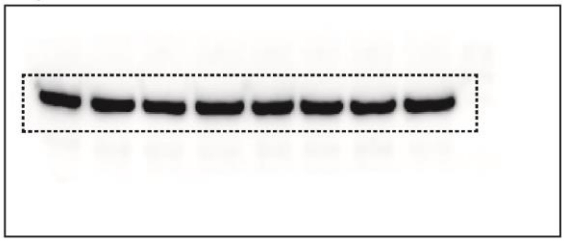

Supplementary Figure-18 (Ford)

Figure 4a (p53)

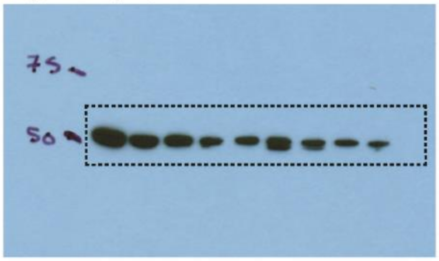

Figure 4a (HDAC1)

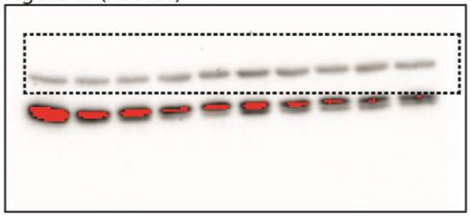

Figure 4b (p53)

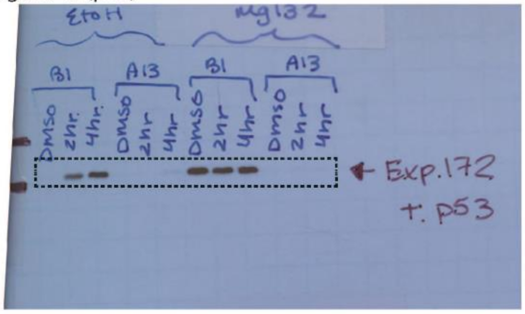

Figure 4b (HDAC1)

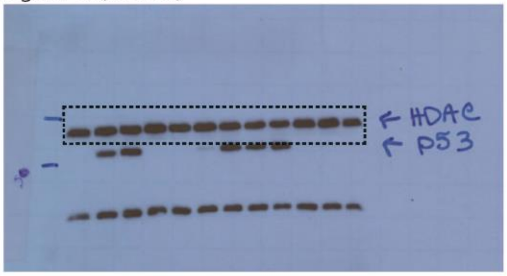

Figure 4b (Cyclin D1)

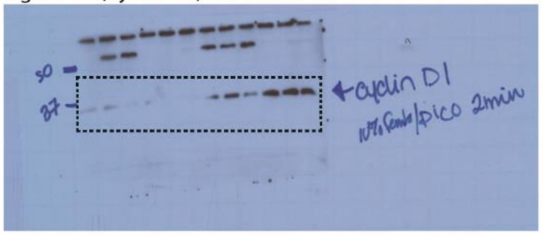

Figure 6b (MCF7 RPL26)

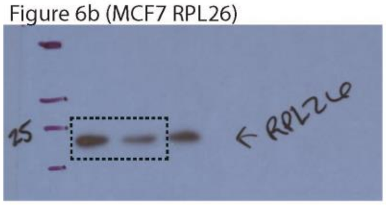

Figure 6b (MCF7 Tubulin)

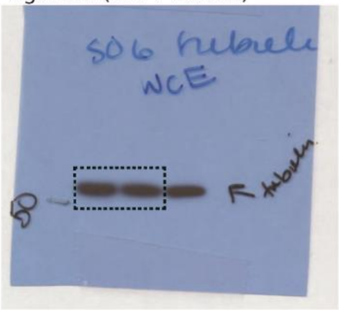

Figure 6b (RKO top- Tubulin, bottom - RPL26)

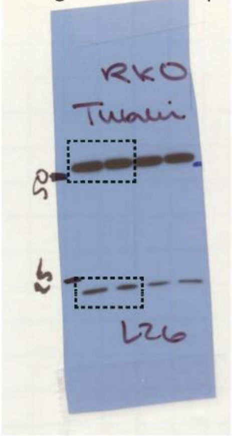

Supplementary Figure-18 (Ford)

Figure 6c (top box-tubulin, bottom box-RPL26)

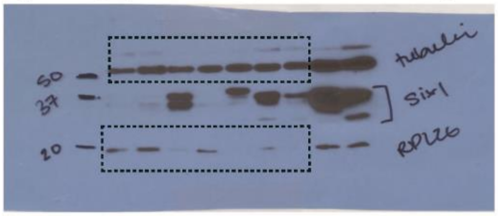

Figure 6c (Six1)

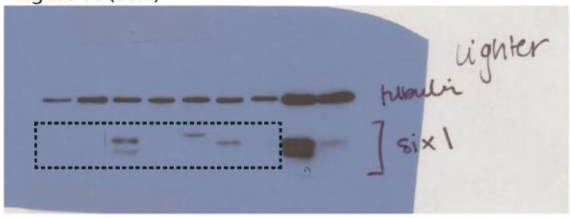

Figure 7c (p53)

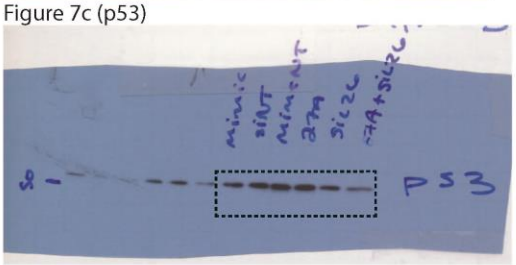

Figure 7c (Runx)

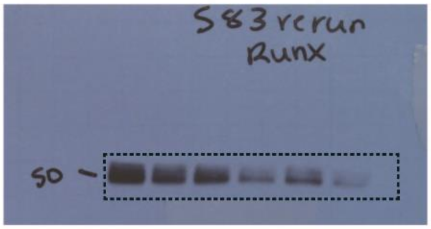

Figure 7c (HDAC1)

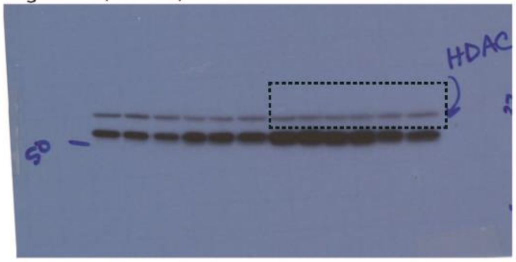

Figure 7c (RPL26)

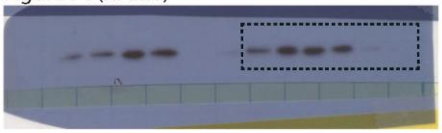

Figure 7c (β-Actin)

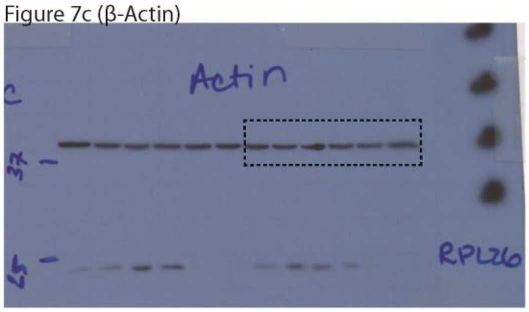

Supplementary Figure-18 (Ford)

Figure 7h (p53)

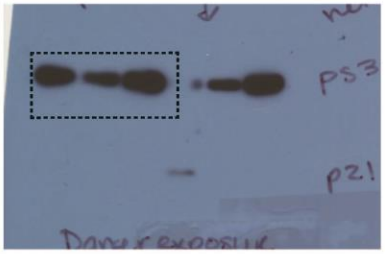

Figure 7h (p21)

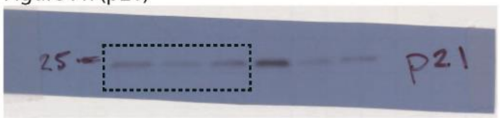

Figure 7h (RPL26)

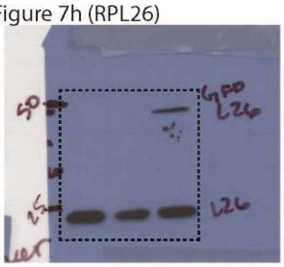

Figure 7h (HDAC1)

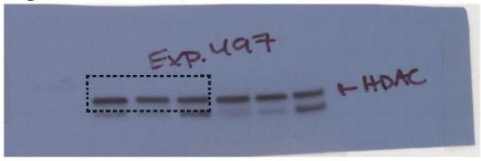

Figure 7h (β-Actin)

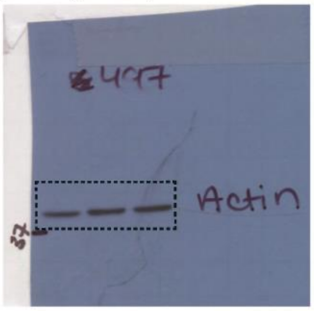

Supplementary Table 1: Summary of patient datasets demonstrating the tumor suppressive associations of RPL26

Tumor Type	Differential Expression	Fold Change	p value	Dataset
Breast	Male breast carcinoma Vs. normal	-2.35	1.1E-22	TCGA breast
Breast	Invasive ductal carcinoma Vs. normal	-2.23	3.71E-41	TCGA breast
Breast	Invasive breast carcinoma Vs. normal	-1.88	2.70E-24	TCGA breast
Breast	Intraductal Cribiform breast adenocarcinoma Vs. normal	-2.42	3.87E-6	TCGA breast
Breast	Invasive lobular breast carcinoma Vs. normal	-1.64	2.45E-12	TCGA breast
Breast	Muscinous breast carcinoma Vs. normal	-1.93	0.004	TCGA breast
Breast	Ductal breast carcinoma in situ epithelia Vs. normal	-2.08	2.43E-6	Ma breast 4
Breast	Ductal breast carcinoma in situ stroma Vs. normal	-1.51	7.69E-5	Ma breast 4
Breast	Invasive ductal breast carcinoma epithelia Vs. normal	-1.78	7.86E-4	Ma breast 4
Breast	Invasive ductal breast carcinoma Vs. normal	-1.72	2.17E-41	Curtis breast
Breast	Invasive breast carcinoma stroma Vs. normal	-15.08	6.94E-16	Finak breast
Bladder	Infiltrating bladder urothelial carcinoma Vs. normal	-1.67	3.35E-6	Lee bladder
Esophageal	Esophageal adenocarcinoma Vs. normal	-3.17	8.87E-13	Kim esophagus
Prostate	Primary site Vs. metastatic site	-5.74	1.80E-9	Chandran Prostate
Prostate	Primary site Vs. metastativ site	-2.78	9.12E-10	Grasso Prostate

p-values were calculated in Oncomine using an independent, two-sample, one-tailed Welch's T-Test.

Supplementary Table 2: small RNA sequences

Target	Target Sequence	Product #	Product name	Company
MDM2 #1	GAUGAGAAGCAACAACAUA	L-003279-00-0005	ON-TARGETplus SMARTpool siRNA	Dharmacon
MDM2 #2	GAAUUUAGACAACCUGAAA	L-003279-00-0005	ON-TARGETplus SMARTpool siRNA	Dharmacon
MDM2 #3	GAACAAGAGACCCUGGUUA	L-003279-00-0005	ON-TARGETplus SMARTpool siRNA	Dharmacon
MDM2 #4	GCCAGUAUAUUAUGACUAA	L-003279-00-0005	ON-TARGETplus SMARTpool siRNA	Dharmacon
Six1 #1	GCCAGGAGCUCAAACUAUU	L-020093-00-0005	ON-TARGETplus SMARTpool siRNA	Dharmacon
Six1 #2	GCACAAGAACGAGAGCGUA	L-020093-00-0005	ON-TARGETplus SMARTpool siRNA	Dharmacon
Six1 #3	GAAGGCGCAUUACGUGGAG	L-020093-00-0005	ON-TARGETplus SMARTpool siRNA	Dharmacon
Six1 #4	GCAGCAAGGCGGAAACCUG	L-020093-00-0005	ON-TARGETplus SMARTpool siRNA	Dharmacon
RPL26 #1	GAUCCAUGCCCAUCCGAAA	L-011132-01-005	ON-TARGETplus SMARTpool siRNA	Dharmacon
RPL26 #2	AAAAGAUCCUCGAACGGAA	L-011132-01-005	ON-TARGETplus SMARTpool siRNA	Dharmacon
RPL26 #3	CCGAAGCAAGAAUCGCAAA	L-011132-01-005	ON-TARGETplus SMARTpool siRNA	Dharmacon
RPL26#4	AGACAGAAGUACAACGUGC	L-011132-01-005	ON-TARGETplus SMARTpool siRNA	Dharmacon
Non-targeting #1	UGGUUUACAUGUCGACUAA	D-001810-10-005	ON-TARGETplus SMARTpool siRNA	Dharmacon
Non-targeting #2	UGGUUUACAUGUUGUGUGA	D-001810-10-005	ON-TARGETplus SMARTpool siRNA	Dharmacon
Non-targeting #3	UGGUUUACAUGUUUUCUGA	D-001810-10-005	ON-TARGETplus SMARTpool siRNA	Dharmacon
Non-targeting #4	UGGUUUACAUGUUUUCCUA	D-001810-10-005	ON-TARGETplus SMARTpool siRNA	Dharmacon
miR-27a	UUCACAGUGGCUAAGUUCCGC	C-300502-03-0010	miRIDIAN mimic	Dharmacon
miR-26b	UUCAAGUAAUUCAGGAUAGGU	C-300501-07-0010	miRIDIAN mimic	Dharmacon
Negative Control	UCACAACCUCCUAGAAAGAGUAGA	CN-001000-01-05	miRIDIAN mimic negative control #1	Dharmacon

Supplementary Table 3: Cell Culture Conditions for each cell line used

Line	Medium	Serum	Additives
MCF7	DMEM high glucose	10% FBS	L-glutamine, Pen/Strep
293T	DMEM high glucose	10% FBS	L-glutamine, Pen/Strep
MCF12A	DMEM/F12	5% HS	insulin, hydrocortisone, EGF, cholera toxin
66Cl4	DMEM high glucose	10% Calf serum	L-glutamine, Pen/Strep, Non-essential amino acids
RKO	RPMI	10% FBS	Pen/Strep, Non-essential amino acids
BT-474	RPMI	10% FBS	Pen/Strep
HCT-116	RPMI	10% FBS	Pen/Strep, Non-essential amino acids

*Cell lines have been cultured long term in the Ford lab, and in the case of RKO, HCT-116 and BT-474 were obtained from John Tentler and Peter Kabos both at the University of Colorado. All cell lines were profiled via short tandem repeat (STR) profiling to confirm their identity.

Supplementary Table 4: Drugs used in-vitro

Drug	Company	Location	Product Number
Nutlin-3	Calbiochem	Billerica, MA	444151
JNJ26854165	Selleck Chemicals	Houston, TX	S1172
MG-132	Calbiochem	Billerica, MA	474791
Cycloheximide	Calbiochem	Billerica, MA	239765
Etoposide	Sigma-Adridge	St. Louis, MO	E1383

Supplementary Table 5: Antibodies used for western blotting, Immunocytochemistry, and ChIP

Antibody	Company	Location	Product Number	Dilution/Concentration used
AlexaFluor 594 α-mouse IgG	Invitrogen	Grand Island, NY	A11032	1:200
AlexaFluor 680 α-rabbit IgG	Invitrogen	Grand Island, NY	A21109	1:200
ATM	Cell Signaling	Danvers, MA	2873	1:1000
β-Actin	Sigma-Adridge	St. Louis, MO	A5316	1:10,000
BrdU	Roche	New York, NY	11170376	1:10
Cyclin D1	Santa Cruz	Dallas, TX	246	1:1000
HDAC1	Santa Cruz	Dallas, TX	7872	1:1000
MDM2	Calbiochem	Billerica, MA	OP46	1:1000
p21	Cell Signaling	Danvers, MA	2946	1:1000
Cleaved Parp	BD Biosciences	San Jose, CA	51-8111KC	1:1000
Total p53	Santa Cruz	Dallas, Texas	6243	1:1000
p53 DO-1	Santa Cruz	Dallas, Texas	126	1:1000
P53 DO-1	Calbiochem	Billerica, MA	OP43	0.5μg
s15-p53	Cell Signaling	Danvers, MA	9286	1:1000
RPL26	Cell Signaling	Danvers, MA	2065	1:1000
Runx1	Epitomics	Burlingame, CA	2593-1	1:1000
Normal mouse IgG	Santa Cruz	Dallas, TX	2025	0.5μg
HRP-anti-mouse IgG	Sigma-Adridge	St. Louis, MO	A9044	1:10,000
HRP-anti-rabbit IgG	Sigma-Adridge	St. Louis, MO	A9169	1:10,000

Supplementary Table 6: Primer sequences used for qRT-PCR

Primer Name	Forward Sequence	Reverse
TP53	TCAACAAGATGTTTTGCCAACTG	ATGTGCTGTGACTGCTTGTAGATG
P21	CTGGAGACTCTCAGGGTCGAAA	GATTAGGGCTTCCTCTTGGAGAA
HDM2	GGCGATTGGAGGGTAGACCT	CACATTTGCCTGGATCAGCA
Six1	TGCGCCGAAAATTTCCA	TTGAAGCAGTAGCTGGTCTCC
PPIB	GGAGATGGCACAGGAGGAAA	CGTAGTGCTTCAGTTTGAAGT
GAPDH	CATCACCATCTTCCAGGAGC	ATGCCAGTGAGCTTCCCGTC
RPL26	AGCGGGTGTAATCAGCAGCC	TCGAGTTCCCAAATCCCCGG
U6	CGCAAGGATGACACGCAAATTCGTGA	Universal Reverse Primer (Qiagen)
GDF15	TAACCAGGCTGCGGGCCAAC	CAGCCGCACTTCTGGCGTGA
BAX	TGGAGCTGCAGAGGATGATTG	AAACATGTCAGCTGCCACTCG
PIG3	AAATTCACCAAAGGTGCTGGAGT	TCCGCCTATGCAGTCTAGAATAAGA
BTG2	GCGCTCCAGGAGGCACT	GGACGGCTTTTCGGGAA
14-3-3σ	GCCGAACGCTATGAGGACAT	CTTCTCCACGGCGCCTT
DDB2	TCTACTCGCTGCCGCACAGG	TCGGGACTGAAACAAGCTGCGT
DR5	TGTCGCCGCGGTCCT	TGGGTGATCAGAGCAGACTCAG
P21 promoter (-2283)	AGCAGGCTGTGGCTCTGATT	CAAAATAGCCACCAGCCTCTTCT
P21 promoter (-1391)	CTGTCCTCCCCGAGGTCA	ACATCTCAGGCTGCTCAGAGTCT
P21 promoter (+11443)	TCTGTCTCGGCAGCTGACAT	ACCACAAAAGATCAAGGTGAGTGA
